# Weight control interventions improve therapeutic efficacy of dacarbazine in melanoma by reversing obesity-induced drug resistance

**DOI:** 10.1186/s40170-016-0162-8

**Published:** 2016-12-07

**Authors:** Parmanand Malvi, Balkrishna Chaube, Shivendra Vikram Singh, Naoshad Mohammad, Vimal Pandey, Maleppillil Vavachan Vijayakumar, Revathy Meenatheril Radhakrishnan, Muralidharan Vanuopadath, Sudarslal Sadasivan Nair, Bipin Gopalakrishnan Nair, Manoj Kumar Bhat

**Affiliations:** 1 Laboratory No. 6, National Centre for Cell Science (NCCS), Savitribai Phule Pune University Campus, Ganeshkhind, Pune, 411 007 India; 2 Amrita School of Biotechnology, Amrita Vishwa Vidyapeetham, Clappana P.O., Kollam, 690 525 India; 3 Present address: Laboratory of Neuroscience, Department of Biotechnology and Bioinformatics, Hyderabad Central University, Hyderabad, 500 046 India

**Keywords:** Obesity, Adipokines, Melanoma, Chemotherapy, Orlistat, Weight-control interventions

## Abstract

**Background:**

Obesity-related cellular, metabolic, and molecular alterations have been shown to increase cancer risk and tumor progression and are associated with poorer therapeutic outcome in cancer patients. However, the impact of obesity and weight-control interventions on the therapeutic response in melanoma is poorly understood.

**Methods:**

High fat diet (HFD)-induced obese mouse model was used in this study to evaluate the outcome of dacarbazine (DTIC) therapy in melanoma. We employed LC-MS/MS to determine the quantity of the drug in tumor, and in various tissues. Unique in vitro approach was used to complement in vivo findings by culturing melanoma cells in either conditioned medium (CM) obtained from differentiated adipocytes or in serum collected from experimental mice.

**Results:**

We report that diet-induced obesity impairs the outcome of DTIC therapy and reduces overall survival in tumor-bearing mice. We provide evidence that obesity restricts the accessibility of DTIC to tumor tissue. Critically, upon curtailing adiposity, accumulation and efficacy of DTIC is significantly improved. Moreover, using appropriate in vitro approaches, we show that melanoma cells exhibit a drug-resistant phenotype when cultured in serum collected from diet-induced obese mice or in CM collected from 3T3-L1 adipocytes. The impaired therapeutic response to DTIC in obese state is mediated by fatty acid synthase (FASN), caveolin-1 (Cav-1), and P-glycoprotein (P-gp). The response to DTIC and overall survival were improved upon employing weight control interventions in the tumor-bearing HFD-fed (obese) mice.

**Conclusions:**

This study indicates that obesity not only supports rapid melanoma progression but also impairs the outcome of chemotherapy, which can be improved upon employing weight control interventions. From clinically relevant point of view, our study exemplifies the importance of lifestyle interventions in the treatment of obesity-promoted cancers.

**Electronic supplementary material:**

The online version of this article (doi:10.1186/s40170-016-0162-8) contains supplementary material, which is available to authorized users.

## Background

Obesity, owing to excess adiposity, is associated with increased risk of many cancer types [1–3]. With the unprecedented surge in global epidemic of obesity and overweight population, incidences of obesity-associated health complications are also likely to increase [4, 5]. Adiposity-related cellular, metabolic, and molecular alterations are known to promote cancer risk and rapid tumor progression [3–7] and adversely affect the response to cancer therapy [8]. However, the positive aspects of obesity management and weight control interventions on the outcome of chemotherapy are not well understood.

Adipose tissue, an endocrine organ, is considered as one of the critical factors involved in tumor progression, angiogenesis, invasion, and metastasis [9–13]. Adipose tissue expansion under persistent nutritional load alters serum profile of adipokines, cytokines, and lipids [7, 10]. These changes eventually create a state of chronic low-grade inflammation [14–16], which supports cancer cell survival and proliferation [17, 18]. Adiposity in overweight and obese people leads to dysregulation of adipocyte functions and associated pathogenesis. Poor survival of cancer patients due to adiposity is an emerging, yet under explored, issue of clinical significance. Meta-analysis of observational studies has established a strong relationship between development and progression of cancers including melanoma, with obesity [1–3]. Melanoma, a life-threatening malignancy which arises in the vicinity of subcutaneous adipose tissue, accounts for the majority of skin cancer-associated mortalities [19]. Obesity affects diagnosis and treatment because of its interference with medical imaging, lowering of tumor markers expression, alteration in the pharmacokinetics of chemotherapeutic drugs, compromised immune surveillance, improper precision of radiotherapy, and increased risk of surgical complications and recovery time [20–22]. In addition, tumor type and degree of adiposity add up to the complexity of therapy and further restrict the choices of chemotherapeutic drugs under obesity [20]. Contribution of adipocytes to chemoresistance of melanoma involves multiple signaling pathways and, hence, is difficult to target them all [23].

Dacarbazine (DTIC) is the only US FDA-approved cytotoxic drug available for the treatment of metastatic melanoma with overall response rate of mere 20–25% [24]. Owing to its proximity to adipose tissue, under obesity, melanoma assumes aggravated lethality with accompanying drug resistance, which makes this association intriguing. However, administration of DTIC at doses based on weight or body size is a complicated medical issue which may have adverse effects [25]. In contrast, any discretion to decrease chemotherapy dose as in normal practice may lead to exposure of cancer cells to a non-effective dosage of the drug that potentially contributes to drug resistance. Therefore, implications of strategy to control the proliferation and invasiveness of cancer in the background of obesity by containing adiposity need to be tested in a preclinical setup before being explored clinically.

Previously, we reported that while diet-induced obesity promotes melanoma growth [26], pharmacological and dietary interventions targeting obesity reverse it to a great extent [27]. Clinically, it has been proposed that a combination of changes in lifestyle together with pharmacological approaches could be a more effective strategy for the management of obesity-promoted cancers [28–30]. In addition, unlike their relatively lean counterparts, the obese cancer patients require specific dosing for a curative response to treatment and overall survival [25]. On these lines, we hypothesized that weight control interventions in conjunction with cancer chemotherapy could have a significant positive impact on the management of obesity-promoted cancers. By implicating pharmacological and dietary interventions to contain adiposity, we have explored the therapeutic outcome of DTIC in melanoma using appropriate in vitro and in vivo models.

## Methods

### Experimental animals and diets

C57BL/6J mice were procured from Experimental Animal Facility (EAF) at National Centre for Cell Science (NCCS), Pune, India. High fat diet (24% fat) was purchased from Provimi Animal Nutrition Pvt. Ltd., Bangalore, India, and normal diet (5% fat) was obtained from Amrut Laboratory, Pune, India. The compositions of diets are provided in supplementary data (Additional file 1: Table S1). Diet-induced obesity was developed in the mice by feeding them with high fat diet as described previously [26, 27]. Male C57BL/6J mice (6–8 weeks old) were divided into normal diet (ND) and high fat diet (HFD) group. ND group was fed with normal diet while HFD group was fed with high fat diet supplemented with ground nut and dried coconut for 6 months. Body weight and serum chemistry profile were measured monthly to verify obesity-associated changes. Water and food were provided ad libitum to all the mice. All animal experiments were carried out as per the requirement and guidelines of the Committee for the Purpose of Control and Supervision of Experiments on Animals (CPCSEA), Government of India, and after obtaining permission of the Institutional Animal Ethics Committee (IAEC).

### Cells and culture conditions

Murine melanoma cells B16F10 and B16F1 and murine pre-adipocyte cells 3T3-L1 were procured from American Type Culture Collection (ATCC, Manassas, VA, USA) and maintained at our in-house cell repository at National Centre for Cell Science, Pune, India. Cells were routinely cultured in Dulbecco’s Modified Eagles Medium (DMEM) supplemented with 10% heat inactivated fetal bovine serum (Hyclone, UT, USA or Gibco, NY, USA), penicillin (100 U/ml), and streptomycin (100 μg/ml) (Invitrogen Life Technologies, CA, USA) and maintained at 37 °C in a 5% CO_2_ humidified incubator (Thermo Fisher Scientific, OH, USA).

### Serum biochemical analysis

Blood glucose levels and serum lipids were estimated as described previously [26, 27]. Insulin, leptin, and adiponectin levels in the serum were estimated by mouse-specific respective ELISA kits as described [23]. Resistin, IL-6, and TNF-α levels in the serum were detected by indirect ELISA as described previously [27].

### Orlistat treatment and/or diet shifting in HFD mice, tumor challenge, DTIC administration, and follow-up

**Fig. 1:**
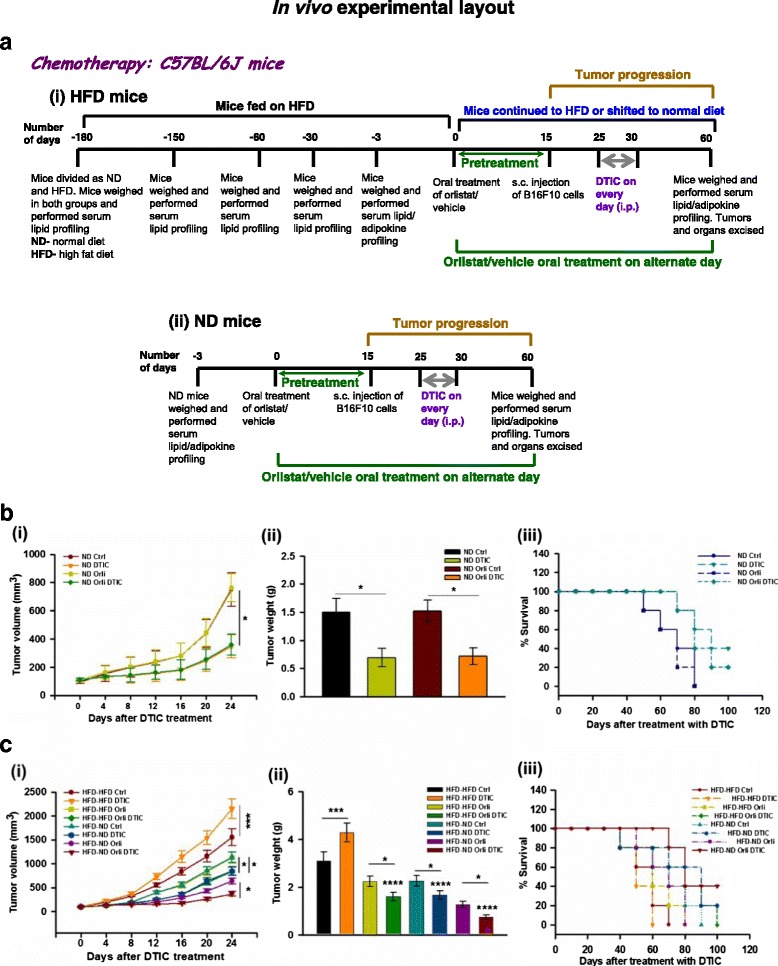
Diet-induced obesity impairs the outcome of DTIC therapy in melanoma which is improved upon employing weight control interventions. **a** Impact of diet-induced obesity on the outcome of DTIC therapy in melanoma isografted HFD C57BL/6J mice (*i*). Effect of treatment of orlistat, at antiobesity dose (10 mg/kg, oral), on the outcome of DTIC therapy in melanoma isografted ND C57BL/6J mice (*ii*). **b** ND male C57BL/6J mice were divided into two major groups. One group was orally treated with orlistat (10 mg/kg), and the other group was treated with vehicle control on every alternate day for 8 weeks. Both the groups of mice (*N*= 11 per each group) were injected with B16F10 cells (2 × 10^5^ cells/mouse in 100 μl PBS). After tumor formation, vehicle or DTIC treatment was given as per the experimental layout shown in Fig. 1. (*i*) Tumor volume, (*ii*) tumor weight, and (*iii*) survival of tumor-bearing mice (*N* = 5 mice per group). **c** HFD male C57BL/6J mice were divided into two major groups. One group was continuously fed with HFD, whereas the other group was shifted from HFD to ND. Mice from both the groups were orally treated with orlistat (10 mg/kg) or vehicle control on every alternate day for 8 weeks. All the groups of mice (*N* = 11 per each group) were injected with B16F10 cells (2 × 10^5^ cells/mouse in 100 μl PBS). After tumor formation, vehicle or DTIC treatment was given as per the experimental layout shown in Fig. 1. (*i*) Tumor volume, (*ii*) tumor weight, and (*iii*) survival of tumor-bearing mice (*N* = 5 mice per group). The results are given as means ± standard deviation; *p < 0.05, **p < 0.01, ***p < 0.001, and ****p < 0.0001 denote significant differences between the groups; *NS* non-significant

To study the impact of diet-induced obesity on the outcome of DTIC therapy in melanoma, we treated B16F10 melanoma isografted obese mice with DTIC purchased from Sigma, USA (80 mg/kg intraperitoneally for five consecutive days as described previously) [31]. In addition, to look into the impact of weight loss interventions on the outcome of DTIC therapy in obese mice, mice were administered with orlistat (an antiobesity drug known to inhibit gastrointestinal lipases) and/or shifted from high fat to normal diet followed by DTIC treatment. The detailed experimental plan is illustrated in Fig. 1a. Briefly, obese mice were administered orally with orlistat (10 mg/kg on every alternate day) purchased from Enzo Life Sciences, NY, USA, and/or shifted from high fat diet to normal diet. HFD C57BL/6J mice treated with vehicle or orlistat were termed as HFD-HFD Ctrl and HFD-HFD Orli (*N* = 11 per each group), respectively, whereas HFD C57BL/6J mice shifted to normal diet and treated with vehicle or orlistat were grouped as HFD-ND Ctrl and HFD-ND Orli (*N* = 11 per each group), respectively. After 15 days, these mice were injected subcutaneously (s.c.) with B16F10 cells (2 × 10^5^) in 100 μl of PBS and monitored daily for the presence of palpable tumors and dimensions were recorded on alternate days. When tumor size became approximately 40–50 mm^3^, all groups of mice were treated with vehicle (acidified water) or DTIC as mentioned above. Tumor volume was calculated using the formula 0.52 × length × width^2^ and was followed up throughout the study. At the end of the experiment, mice were sacrificed by CO_2_ euthanasia. Excised tumors’ volume and weight were recorded, and the samples were immediately preserved at −80 °C until further use. To observe their survival rates, five mice from each group were followed up for an additional 60 days.

### Orlistat treatment in ND mice, tumor challenge, DTIC administration, and follow-up

For investigating whether oral delivery of orlistat influences DTIC treatment in melanoma-bearing ND C57BL/6J mice, these mice were divided in to two major groups (*N* = 11 per each group): ND mice treated with vehicle or orlistat 15 days prior to injecting melanoma cells were termed as (i) ND Ctrl and (ii) ND Orli, respectively. Melanoma cells were injected and orlistat treatment was continued. Following tumor formation, all groups of mice were treated with vehicle (acidified water) or DTIC, and tumor volume was followed up in these mice throughout the study as mentioned above. The detailed experimental plan has been illustrated in Fig. 1a (ii). At the end of the experiment, mice were sacrificed, tumors were excised, and the samples were immediately preserved at −80 °C until further use. Five mice from each group were followed up for survival studies as mentioned above.

### Immunoblotting

Melanoma tumor samples or cells were washed three to five times with ice-cold PBS and lysed in ice-cold RIPA lysis buffer as described previously [27]. Briefly, the samples were centrifuged at 12000 RPM for 40 min and clear supernatants were stored at −80 °C. Protein concentrations were determined by Coomassie Plus Protein Assay Reagent (Thermo Scientific, IL, USA). Equal amounts of protein samples (50–100 μg) were resolved on 8–10% SDS-polyacrylamide gel and then transferred onto PVDF membrane (Millipore, MA, USA). The membranes were blocked and further probed with antibodies against caveolin-1 (Cav-1) (1:1000), fatty acid synthase (FASN) (1:1000), pAkt (Ser-473) (1:1000), total Akt (1:1000), PCNA (1:1000), cyclin D1 (1:1000), β-tubulin (1:1000) (Santa Cruz Biotechnology, CA, USA), and P-glycoprotein (P-gp) (1:1000) (Abcam, MA, USA). After washing, the membranes were incubated with HRP-conjugated secondary antibodies (1:2000) and blots were developed using luminescence detection reagents (Santa Cruz Biotechnology, CA, USA).

### LC-MS/MS analysis and quantitation of DTIC in tissue extracts

LC-MS/MS analysis was carried out on an Agilent 1290 Infinity ultra-high performance liquid chromatography (UHPLC) system coupled to an Agilent 6540 UHD Accurate Mass Q-TOF mass spectrometer equipped with a Dual AJS electrospray ionization (ESI) source. The samples were introduced to the mass spectrometer through a reversed-phase column (Agilent ZORBAX SB-C18, 2.1 × 30 mm, 3.5 μm). The mobile phase containing water and acetonitrile with 0.1% formic acid was infused at a flow rate of 0.4 ml min^−1^. The MS scan range was set between 100 and 1000 m/z, with the dry gas flow and dry gas temperature maintained at 6 L min^−1^ and 320 °C, respectively. Introduction of reference molecules with m/z, 121.05087, 149.02332, 322.04812, and 922.00979 ensured consistency in mass accuracy over a wide mass range. All the mass spectrometric data were acquired in positive ionization mode using Agilent MassHunter data acquisition software, version B.05.01. MassHunter Quantitative analysis software, version B.07.00 was used for the generation of calibration curves and subsequent quantitation of DTIC in the samples. Calibration curves were created using linear fitting, 1/x weighting and including the origin. Calibration standards (0.5–800 ng) were prepared using standard DTIC. All the samples including the standard DTIC preparations were spiked with leucine enkephalin (10 ng) as internal standard. Extracted ion chromatograms of DTIC (m/z, 183.0989) and leucine enkephalin (m/z, 556.2766) were applied for quantitation. The quantitation capability of this method is evaluated by using samples with known concentrations.

### Culture of melanoma cells in serum and conditioned medium

Approximately 1.5 × 10^2^ B16F10 cells were plated in 24-well plates and allowed to adhere. After 24 h, DMEM containing 5% serum collected from experimental C57BL/6J mice (as illustrated in Fig. 1) was added and cells were cultured chronically for 10 days. Medium was changed on every 2–3 days. Thereafter, cells were fixed with paraformaldehyde and stained with crystal violet, and images were taken using digital camera (Olympus, Tokyo, Japan).

3T3-L1 cells were plated in 35-mm dishes and differentiated as described [32], along with vehicle or orlistat treatment (50 μM). The medium was changed every alternate day and fresh medium containing orlistat or cerulenin (an inhibitor of FASN) was added to the cells. After 11 days, medium was removed and cells were washed twice with DMEM. Fresh DMEM was added and cells were incubated for further 18 h. For culturing the melanoma cells, conditioned medium (CM) was mixed with fresh DMEM in 1:1 ratio. Approximately 3 × 10^2^ B16F10 cells were plated in 12-well plates and cultured chronically for 10 days in this CM. Thereafter, crystal violet staining was performed to verify long-term survival, and the plates were photographed.

### MTT assay

Melanoma cells were plated at a density of 6 × 10^3^ cells/well in 96-well plates and allowed to adhere. After 24 h, cells were treated with vehicle (PBS or ethanol), inhibitors, or drugs as per the experimental requirements. After treatment duration, medium was removed and 50 μl of MTT (methylthiazole tetrazolium, 1 mg/ml in DMEM without phenol red) (Sigma-Aldrich, MO, USA) was added in each well and further incubated for 4 h at 37 °C. Formazan crystals were solubilized in 100 μl of isopropanol, and absorbance was measured at 570 nm.

### Long-term survival assay

Melanoma cells were plated at a density of 3 × 10^2^ cells/well in 12-well plates. Next day, these cells were treated with vehicle or orlistat as per the experimental requirements. After 48 h, medium was removed and fresh medium was added. Cells were allowed to grow for 10 days with medium change on every 2–3 days. Thereafter, cells were fixed with 3% paraformaldehyde for 10 min and stained with 0.05% crystal violet for 2 h at room temperature. Images were taken using digital camera (Olympus, Tokyo, Japan).

### Rhodamine-123 efflux assay

To measure the efflux of Rhodamine-123 (Rh-123), which is reflective of transport activity for P-gp, melanoma cells were seeded at a density of 1 × 10^3^ cells/well in 35-mm culture dishes and allowed to adhere for 24 h. Thereafter, these cells were chronically cultured in DMEM containing serum collected from experimental mice or in CM form 3T3-L1 cells for 10 days with medium change on every 2–3 days. Cells were washed thrice with PBS and incubated for 30 min at 37 °C in PBS containing 2 μM Rh-123. Further, Rh-123 efflux was measured using Flow cytometer. Fluorescence intensity of Rh-123 was acquired using FACS Calibur, and the data were analyzed using CellQuest Pro software (BD Biosciences, CA, USA).

### Immunofluorescence confocal staining

Melanoma cells were plated in multi-well chambered slides (MP Biomedicals, OH, USA) and allowed to grow for 24 h. Next day, medium was changed to DMEM supplemented with serum from ND or HFD mice or in CM collected from 3T3-L1 cells for 10 days. Subsequently, these cells were washed with PBS, and immunofluorescence staining was performed as described previously [26].

### Inhibitor-based in vitro studies

Melanoma cells were seeded at an appropriate density in culture dishes or well plates and allowed to adhere. After 24 h, cells were grown in DMEM containing serum collected from experimental mice or in CM collected from 3T3-L1 cells in the presence of vehicle or pharmacological inhibitors for 48 h. Thereafter, cells were processed for MTT assay or long-term survival assay or Rhodamine-123 efflux analysis as mentioned above.

### Statistical analysis

Statistical analysis was performed using GraphPad Prism 7.0 (GraphPad Software Inc., CA, USA). All data are presented as the mean ± standard deviation (S.D.). For in vitro experiments, bars represent variations within the wells of the experiment. The experiments were repeated at least three times. For in vivo experiments involving more than two groups, one-way ANOVA was used, followed by the Tukey multiple comparison test. In vitro or in vivo data involving two experimental groups were analyzed using two-tailed unpaired Student’s *t* test. The values of *p* < 0.05, *p* < 0.01, *p* < 0.001, and *p* < 0.0001 were considered as statistically significant (*), very significant (**), highly significant difference (***), and very highly significant difference (****), respectively, unless otherwise mentioned.

## Results

### Diet-induced obesity impairs the outcome of DTIC therapy in melanoma

We have reported previously that diet-induced obesity and weight loss interventions influence melanoma progression [26, 27]. Among many, diet is one of the important factors that influence chemotherapeutic response in patients. Therefore, to investigate whether diet-induced obesity or dietary interventions could dictate the outcome of chemotherapy in melanoma, we developed diet-induced obesity by chronic feeding of C57BL/6J mice with high fat diet as described previously [26, 27]. After developing diet-induced obese phenotype, ectopic isografts were induced by injecting B16F10 cells as shown in the experimental layout in Fig. 1a (i). Upon the appearance of palpable tumors, DTIC was administered in these mice and tumor volume and obesity-associated parameters were regularly monitored. Parallely, similar experiment was performed in normal diet-fed (ND) mice (Fig. 1a (ii)). As expected, tumor progression was significantly reduced in ND mice treated with DTIC as compared to control (Fig. 1b (i), (ii)). Additionally, DTIC treatment prolonged the survival of ND mice in comparison to the untreated tumor bearing mice (Fig. 1b (iii)). Surprisingly, enhanced tumor growth in HFD mice treated with DTIC in comparison to their untreated counter parts at the same dosage was observed (Fig. 1b (i), (ii)). In untreated HFD mice, overall survival was reduced when compared to their respective ND counter parts. Moreover, survival in HFD mice was reduced due to increased tumor burden under DTIC treatment (Fig. 1c (iii)). Further, to confirm whether DTIC treatment itself altered obesity-associated parameters in ND and HFD mice, serum chemistry profiles were assessed. We found that DTIC per se did not affect the levels of obesity-associated factors in ND and HFD mice as compared to their respective untreated controls (Table 1 and Additional file 2: Table S2). These results indicate that diet-induced obesity impairs the outcome of DTIC therapy in melanoma.

**Table 1:** Evaluation of obesity-associated factors in HFD C57BL/6J mice

Parameters	C57BL/6J HFD							
	HFD-HFD Ctrl	HFD-HFD DTIC	HFD-HFD Orli	HFD-HFD Orli DTIC	HFD-ND Ctrl	HFD-ND DTIC	HFD-ND Orli	HFD-ND Orli DTIC
Body weight (g)	32.03 ± 1.94	32.28 ± 1.70 (*p* = 0.999)	26.16 ± 0.71 (*p* < 0.0001)	26.48 ± 0.85 (*p* < 0.0001)	27.13 ± 0.75 (*p* < 0.0001)	26.73 ± 1. 3 (*p* < 0.0001)	23.1 ± 1.02 (*p* < 0.0001)	22.9 ± 0.92 (*p* < 0.0001)
Blood glucose (mg/dl)	196.17 ± 6.31	194.50 ± 6.98 (*p* = 0.9997)	146.3 ± 5.99 (*p* < 0.0001)	152.33 ± 5.47 (*p* < 0.0001)	174.5 ± 4.72 (*p* < 0.0001)	177.5 ± 5.05 (*p* = 0.0001)	128.83 ± 6.24 (*p* < 0.0001)	129.67 ± 7.4 (*p* < 0.0001)
Serum TG (mg/dl)	84.02 ± 2.89	84.17 ± 1.15 (*p* = 0.9999)	66.03 ± 3.39 (*p* < 0.0001)	67.17 ± 2.76 (*p* < 0.0001)	68.48 ± 2.25 (*p* < 0.0001)	69.15 ± 2.41 (*p* < 0.0001)	60.53 ± 3.15 (*p* < 0.0001)	61.35 ± 1.28 (*p* < 0.0001)
Serum cholesterol (mg/dl)	120.82 ± 4.20	120.54 ± 4.49 (*p* = 0.9995)	95.75 ± 4.91 (*p* < 0.0001)	97.21 ± 4.22 (*p* < 0.0001)	99.50 ± 2.86 (*p* < 0.0001)	100.27 ± 3. 5 (*p* < 0.0001)	88.64 ± 5.17 (*p* < 0.0001)	89.75 ± 4.3 (*p* < 0.0001)
Serum LDLc (mg/dl)	84.86 ± 2.92	85.01 ± 1.17 (*p* = 0.9999)	66.69 ± 3.42 (*p* < 0.0001)	67.84 ± 2.79 (*p* < 0.0001)	69.17 ± 2.27 (*p* < 0.0001)	69. 84 ± 2.4 (*p* < 0.0001)	63.31 ± 3.13 (*p* < 0.0001)	62.96 ± 1.24 (*p* < 0.0001)
Serum free fatty acids (mM/l)	2.22 ± 0.08	2.20 ± 0.04 (*p* = 0.9993)	1.74 ± 0.09 (*p* < 0.0001)	1.73 ± 0.04 (*p* < 0.0001)	1.81 ± 0.06 (*p* < 0.0001)	1.81 ± 0.05 (*p* < 0.0001)	1.66 ± 0.08 (*p* < 0.0001)	1.64 ± 0.04 (*p* < 0.0001)
Serum leptin (ng/ml)	43.20 ± 2.40	43.11 ± 1.65 (*p* = 0.9999)	33.74 ± 2.47 (*p* < 0.0001)	33.83 ± 2.80 (*p* < 0.0001)	35.19 ± 2.49 (*p* < 0.0001)	34.54 ± 2.3 (*p* < 0.0001)	26.54 ± 1.60 (*p* < 0.0001)	25.58 ± 1.38 (*p* < 0.0001)
Serum adiponectin (ng/ml)	3486 ± 273	3421 ± 198 (*p* = 0.9999)	8204 ± 593 (*p* < 0.0001)	8287 ± 304 (*p* < 0.0001)	5292 ± 362 (*p* < 0.0001)	5133 ± 228 (*p* < 0.0001)	9108 ± 550 (*p* < 0.0001)	9189 ± 322 (*p* < 0.0001)
Serum insulin (μg/l)	0.3983 ± 0.02	0.385 ± 0.016 (*p* = 0.8701)	0.269 ± 0.01 (*p* < 0.0001)	0.275 ± 0.021 (*p* < 0.0001)	0.3066 ± 0.02 (*p* < 0.0001)	0.301 ± 0.02 (*p* < 0.0001)	0.2570 ± 0.01 (*p* < 0.0001)	0.2631 ± 0.01 (*p* < 0.0001)
Serum resistin (ng/ml)	2.25 ± 0.10	2.23 ± 0.11 (*p* = 0.9999)	1.66 ± 0.08 (*p* < 0.0001)	1.60 ± 0.07 (*p* < 0.0001)	1.78 ± 0.08 (*p* < 0.0001)	1.82 ± 0.10 (*p* < 0.0001)	1.19 ± 0.06 (*p* < 0.0001)	1.23 ± 0.06 (*p* < 0.0001)
Serum IL-6 (pg/ml)	45.41 ± 2.45	44.99 ± 1.58 (*p* = 0.9999)	35.45 ± 2.88 (*p* < 0.0001)	35.21 ± 2.31 (*p* < 0.0001)	36.40 ± 2.79 (*p* < 0.001)	35.59 ± 2.38 (*p* < 0.0001)	29.25 ± 2.12 (*p* < 0.0001)	27.12 ± 1.83 (*p* < 0.0001)
Serum TNF-α (pg/ml)	29.44 ± 1.74	28.67 ± 0.99 (*p* = 0.9708)	13.78 ± 1.07 (*p* < 0.0001)	14.35 ± 0.62 (*p* < 0.0001)	15.99 ± 1.73 (*p* < 0.0001)	16.17 ± 1.91 (*p* < 0.0001)	11.48 ± 1.12 (*p* < 0.0001)	10.80 ± 0.79 (*p* < 0.0001)

Evaluation of obesity-associated factors in HFD C57BL/6J mice. Obesity-associated parameters in the experimental HFD mice recorded at the end of the experiment. HFD male C57BL/6J mice were divided into two major groups. One group was continuously fed with HFD, whereas the other group was shifted from HFD to normal diet. Mice from both the groups were orally treated with orlistat (10 mg/kg) or vehicle control on every alternate day for 8 weeks. All the groups of mice (*N* = 11 per each group) were injected with B16F10 cells (2 × 10^5^ cells/mouse in 100 μl PBS). After tumor formation, vehicle or DTIC treatment was given as per the experimental layout shown in Fig. 1. Their body weight was monitored weekly throughout the study, and serum was collected at the end of the experiment. Blood glucose, serum TG, serum cholesterol, serum-free fatty acids, and serum LDLc were measured. Serum factors including leptin, adiponectin, insulin, resistin, IL-6, and TNF-α were estimated by ELISA. The results are given as means ± standard deviation

### Weight control interventions improve the efficacy of DTIC in obese mice

In order to look into whether controlling adiposity has any influence on the tumor progression, HFD mice were subjected to weight loss interventions. We used both pharmacological and diet control procedures as described in our previous study [27]. Following oral administration of orlistat (10 mg/kg on every alternate day) and/or shifting obese mice from high fat to normal diet (HFD to ND), on the 15th day, B16F10 melanoma cells were injected. Subsequently, tumor progression and obesity-associated parameters were regularly monitored till the termination of the experiment (Fig. 1a (i)). Similar experiments were also carried out in ND mice to further verify if orlistat, at an antiobesity dose, affects the efficacy of DTIC (Fig. 1a (ii)).

Firstly, we monitored whether diet/weight control interventions alter obesity-associated parameters. Normalization in the levels of obesity-associated factors was observed upon orlistat treatment or by shifting experimental HFD mice to normal diet (Table 1). Interestingly, in these mice, the levels of obesity-associated factors were found to be close to the levels in ND mice, particularly when orlistat administration was combined with diet shifting (Table 1). However, obesity-associated factors were unaffected in DTIC-treated group as compared to respective untreated controls (Table 1).

Next, we checked if orlistat, at antiobesity dose, influenced the outcome of DTIC therapy in ND mice. Consistent with the results of our previously published study [24], tumor volume and weight were found to be unaltered in ND mice administered with antiobesity dose of orlistat, as compared to control mice (Fig. 1b (i), (ii)). DTIC significantly decreased tumor volume and weight in ND mice. However, no further change in the tumor growth was observed in the mice receiving orlistat and DTIC as compared to DTIC alone. Similarly, orlistat treatment did not influence overall survival of mice as compared to mice receiving only vehicle (Fig. 1b (i–iii)). Also, no significant change in the overall survival was observed in mice receiving DTIC alone or mice receiving DTIC and orlistat together (Fig. 1b (i–iii)). Thus, orlistat neither altered the tumor-reducing effect of DTIC nor improved survival in ND mice. Moreover, levels of obesity-associated factors were also not altered (Additional file 1: Table S1), suggesting that orlistat per se does not influence the response to DTIC in ND mice. Interestingly, in tumor-bearing HFD mice, we observed that weight control interventions by orlistat treatment or diet shifting not only restricted tumor growth but also improved the efficacy of DTIC, as is evident by reduction in tumor volume and weight (Fig. 1c (i), (ii)), and survivability increased by 40% (Fig. 1c (iii)). Furthermore, DTIC treatment was much more effective in retarding tumor growth and in improving overall survival when orlistat administration was combined with dietary intervention (80% survival at day 60 in case of orlistat treatment and 100% upon combining diet and orlistat) (Fig. 1c (i–iii)). Survivability of tumor-bearing mice receiving orlistat and DTIC therapy or diet control together with orlistat and DTIC was prolonged up to 90 and 100 days, respectively, in comparison with 60 days for HFD mice receiving DTIC only. These observations were in parallel with normalization in obesity-associated parameters in HFD mice, suggesting that reducing adiposity by weight loss interventions profoundly improves the efficacy of DTIC and increases overall survival of tumor-bearing mice.

### Elevated levels of tumor-promoting molecules and rapid efflux of DTIC contribute to impaired response

**Fig. 2:**
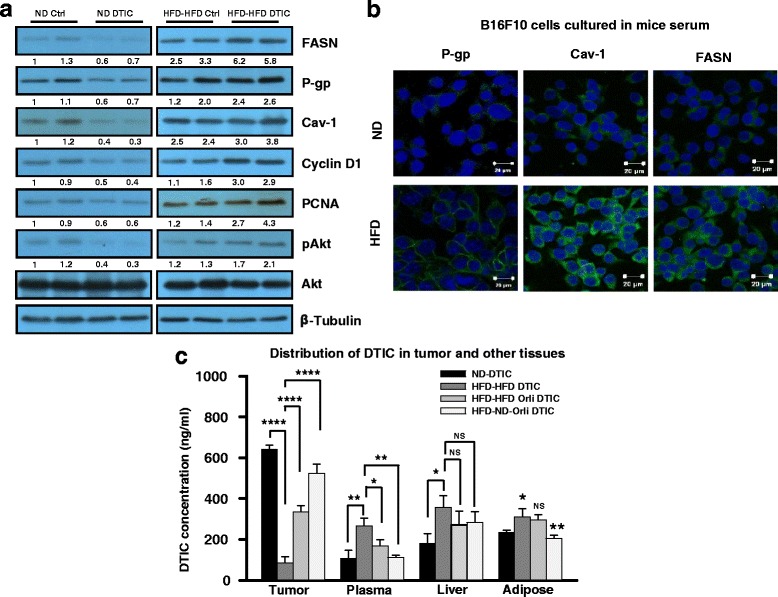
Molecular events associated with the impaired response to DTIC therapy in tumors of the experimental B16F10 isografted mice. **a** Western blot analysis of lysates from tumors of experimental HFD or ND mice subjected to SDS-PAGE and probed for levels of FASN, P-gp, Cav-1, pAkt, PCNA, and cyclin D1 in ND or HFD C57BL/6J mice treated with or without DTIC. **b** B16F10 or B16F1 cells were chronically grown in medium containing 5% serum collected from ND or HFD C57BL/6J mice for 15 days. Thereafter, these cells were subjected to immunofluorescence confocal staining of the indicated molecules (*scale bar* = 20 μm). **c** Quantitative determination of DTIC concentration in the tumor, plasma, liver, and adipose tissues excised from the indicated group of mice. Level of DTIC was determined by mass spectrometric analysis. The results are given as means ± standard deviation; *p < 0.05, **p < 0.01, ***p < 0.001, and ****p < 0.0001 denote significant differences between the groups; *NS* non-significant

To get mechanistic insights, we sought to explore the molecular events contributing to impaired response to DTIC, in diet-induced obesity. To understand as to why DTIC treatment leads to enhanced tumor growth in obese mice, we checked the levels of molecules involved in tumor progression under obese state. Fatty acid synthase (FASN) is a key molecule associated with increased proliferation, survival, and drug resistance in various cancer cells including melanoma. We have earlier reported that increased level of FASN and caveolin (Cav)-1 contributes to the enhanced tumor growth in obese mice [26]. In the present study, we found that FASN level was further increased and Cav-1 levels was also elevated in tumors of HFD mice treated with DTIC (Fig. 2a). On the contrary, as anticipated, FASN and Cav-1 level was much lower in ND mice treated with DTIC (Fig. 2a). Additionally, to verify whether enhancement in the tumor growth is because of increase in proliferation of melanoma cells following DTIC treatment in obese mice, we checked the level of proliferative molecules. It was found that DTIC treatment caused increase in protein levels of cyclin D1 and proliferating cell nuclear antigen (PCNA) in tumors from HFD mice (Fig. 2a). In contrast, reduced levels of these proteins were detected in tumors of ND mice treated with DTIC (Fig. 2a). Changes in level of these molecules were associated with concomitant alteration in the phosphorylation of Akt, an important kinase involved in cell growth, survival, proliferation, and other vital cellular functions [33].

Furthermore, we explored the molecular events those might be involved in mediating impaired therapeutic outcome of DTIC under obese background. We speculated that, because of increased expression of P-glycoprotein (P-gp), tumor cells are not able to retain sufficient quantity of DTIC. This would cause hindrance in the accumulation of an effective concentration of drug in cells. P-gp is a multidrug resistance protein associated with pumping out drugs from the resistant cells [34]. Therefore, we checked the level of P-gp in the tumors of HFD mice administered with or without DTIC. Level of P-gp, which was found to be elevated in tumors of HFD mice, was further increased in tumors from DTIC-treated HFD mice. Under similar setup, DTIC treatment in ND mice reduced the level of P-gp (Fig. 2a). Immunofluorescence staining confirmed the increased expression and localization of P-gp to plasma membrane in B16F10 and B16F1 cells grown in HFD serum as compared to cells cultured in ND serum of C57BL/6J mice (Fig. 2b and Additional file 3: Figure S1, respectively).

To confirm the presence of DTIC in vivo, we checked the distribution of DTIC in tumors and other vital organs by mass spectrometry. We observed significantly reduced level (~6-fold less) of DTIC in tumors excised from HFD mice as compared to the level in ND counterparts (Fig. 2c). DTIC level in the plasma, liver, and adipose tissue from HFD mice was higher as compared to ND mice (Fig. 2c). Concentration of DTIC was found to be even lesser in tumors than in other tissues excised from HFD mice (Fig. 2c). Interestingly, obesity control interventions significantly improved accumulation of DTIC in tumors from HFD mice with concomitant decrease in amount of DTIC in the plasma, liver, and adipose tissue (Fig. 2c). Collectively, these results suggest that increased levels of FASN, Cav-1, and P-gp in tumors are associated with increased tumor growth and impairment in the outcome of DTIC therapy in melanoma under obese state.

### FASN, Cav-1, and P-gp are involved in impaired response of melanoma to DTIC treatment under obesity

**Fig. 3:**
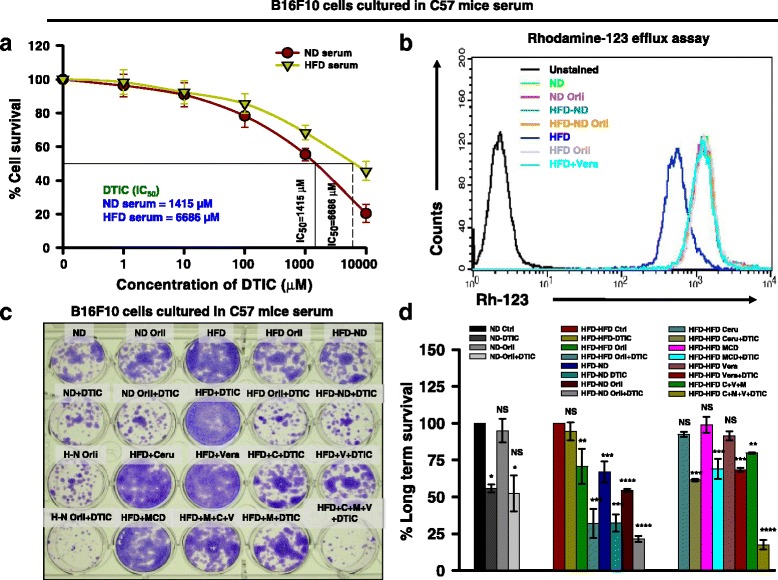
Effect of obesity-associated serum factors on survival of B16F10 cells upon DTIC treatment. **a** Calculation of IC_50_ value of DTIC in DMEM containing ND or HFD serum. B16F10 cells were chronically grown in medium containing 5% serum collected from ND or HFD C57BL/6J mice for 10 days. These cells were then subjected to DTIC treatment at the indicated concentrations for 48 h. Thereafter, MTT assay was performed. **b** Rh-123 efflux assay in B16F10 cells cultured in the medium containing serum from experimental mice. Data were acquired on FACS Calibur and analyzed using BD CellQuest Pro software. **c**, **d** Long-term survival assay in B16F10 cells cultured in the medium containing serum from experimental mice in the presence of inhibitors of FASN, Cav-1, and P-gp, either alone or together with DTIC (**c**). Data were quantitated using ImageJ software (**d**). The data are representative of experiments performed three times; Ceru or C = cerulenin; MCD or M = methyl β-cyclodextrin; Vera or V = verapamil. The results are given as means ± standard deviation; *p < 0.05, **p < 0.01, ***p < 0.001, and ****p < 0.0001 denote significant differences between the groups; *NS* non-significant

To corroborate in vivo findings in vitro, we cultured B16F10 cells in serum collected from ND and HFD C57BL/6J mice. These cells were treated with varying concentrations of DTIC. Similar to in vivo findings, we observed that B16F10 cells cultured in the medium containing HFD serum showed an impaired response to DTIC (Fig. 3a) and inhibitory concentration (IC_50_) of DTIC was significantly increased (~5-fold more) as compared to B16F10 cells cultured in medium containing ND serum (Fig. 3a). The IC_50_ of DTIC (1415 μM) for cells cultured in medium containing ND serum was used for all in vitro experiments performed on cells cultured in serum collected from the experimental mice. Further, to verify the functional status of P-gp in obese state, we performed rhodamine 123 (Rh-123) efflux assays. In B16F10 and B16F1 cells cultured chronically in HFD serum, Rh-123 efflux was higher as compared to those cultured in ND serum (Fig. 3b and Additional file 4: Figure S2, respectively). Moreover, Rh-123 efflux was reduced in cells cultured in medium containing serum from orlistat-treated and/or diet-shifted mice (Fig. 3b and Additional file 4: Figure S2), which correlates with normalized serum levels of obesity-associated factors. The increase in Rh-123 efflux in the cells grown in medium supplemented with HFD serum was reversed upon treatment with verapamil, an inhibitor of P-gp, (Fig. 3b and Additional file 4: Figure S2) confirming the involvement P-gp in the impaired response to DTIC in melanoma under obese state.

Consistent with above stated findings, impairment in the response of B16F10 and B16F1 cells cultured in HFD serum condition to DTIC, in a long-term survival assay was very clearly visible. In B16F10 and B16F1 cells cultured in medium containing serum from orlistat-treated or diet-shifted obese mice, cell survival was reduced by approximately 70 and 75% respectively, upon DTIC treatment (Fig. 3c, d and Additional file 5: Figure S3). Survival was drastically reduced in the cells cultured in medium containing serum from orlistat-treated or diet-shifted obese mice upon treatment with DTIC, which was comparable with the response to DTIC, in ND serum cultured cells (Fig. 3c, d and Additional file 5: Figure S3).

Next, to ascertain the involvement of FASN, Cav-1, and P-gp in the impaired response to DTIC in B16F10 cells cultured in the presence of serum from HFD and ND mice, long-term clonogenic survival assay was performed in the presence or absence of inhibitors of these molecules. We observed that inhibition of these molecules individually increased the cytotoxicity of DTIC in cells cultured in medium containing HFD serum. Moreover, sensitivity of B16F10 and B16F1 cells to DTIC was greatly increased (approximately 80%) when all the three molecules were simultaneously inhibited (Fig. 3c, d and Additional file 5: Figure S3). Therefore, it is likely that FASN, Cav-1, and P-gp are cumulatively responsible for impairment in the outcome of chemotherapy in melanoma.

### Adipocyte-secretory factors impair the response of melanoma to DTIC

**Fig. 4:**
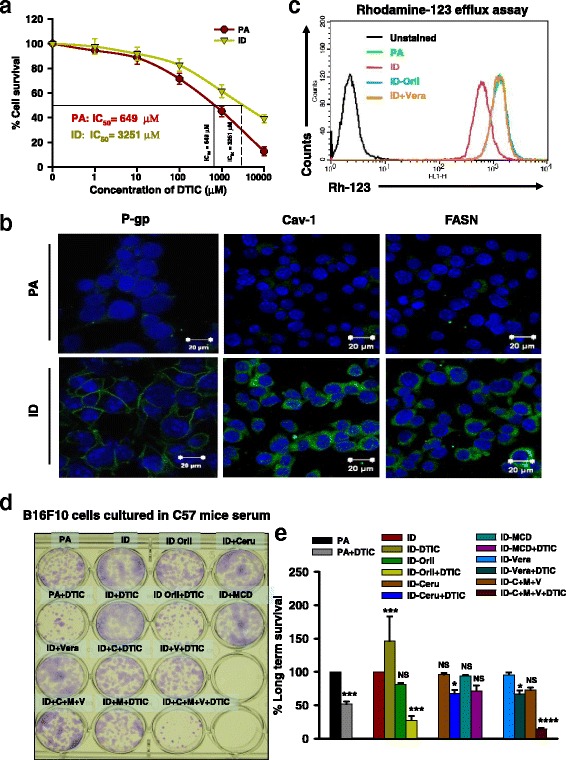
Adipocyte-secreted factors impair the response of melanoma cells to DTIC. **a** Calculation of IC_50_ value of DTIC in CM from 3T3-L1 cells. B16F10 cells were chronically grown in conditioned medium (CM) collected from 3T3-L1 cells for 10 days. These cells were then subjected to DTIC treatment at the indicated concentrations for 48 h. Thereafter, MTT assay was performed. **b** Immunofluorescence confocal staining of P-gp, Cav-1, and FASN in B16F10 cells cultured in CM collected from 3T3-L1 cells (*scale bar* = 20 μm). **c** Rh-123 efflux assay in B16F10 cells cultured in the CM from 3T3-L1 cells. **d**, **e** Long-term survival assay in B16F10 cells cultured in the CM from adipocytes in the presence of inhibitors of FASN, Cav-1, and P-gp, either alone or together with DTIC (**d**). Data were quantitated using ImageJ software (**e**). The data are representative of experiments performed three times; PA = preadipocytes; ID = differentiated adipocytes; Orli = orlistat; Ceru or C = cerulenin; MCD or M = methyl β-cyclodextrin; Vera or V = verapamil. The results are given as means ± standard deviation; *p < 0.05, **p < 0.01, ***p < 0.001, and *****p < 0.0001 denote significant differences between the groups; *NS* non-significant

Above stated observations suggest that controlling obesity was associated with improved response to DTIC in obese mice, which in turn, implies an inter-relationship between normalization in the obesity-associated factors and cellular sensitivity. Melanoma cells grown in the serum collected from obese mice showed poor response to DTIC while reverse was obtained for cells grown in the serum collected from either orlistat treated or diet shifted. Therefore, we speculated that poor response to the DTIC therapy in melanoma under obesity might be influenced by obesity-associated secretory factors. Thus, we explored the role of adipocyte-secreted factors towards the response of melanoma cells to DTIC. To consolidate our observations, we utilized 3T3-L1 cells, which are considered as in vitro model for adipocyte-secreted factors. For this, 3T3-L1 cells were induced to differentiate in the presence or absence of orlistat (an inhibitor of adipocyte differentiation) for 11 days, and the conditioned medium (CM) from these cells was collected. B16F10 cells were cultured in CM and treated with varying concentrations of DTIC. As expected, we noticed that B16F10 cells cultured in CM from differentiated adipocytes showed impairment in the response to DTIC (Fig. 4a). Cells grown in the CM from differentiated adipocytes required much higher concentration (~5-fold more) of drug to achieve 50% cell killing as compared to those grown in the CM of undifferentiated/preadipocytes (Fig. 4a), suggesting that adipocyte secretary factors indeed contribute towards poor response of melanoma cells to DTIC under obese state. IC_50_ value of DTIC (649 μM) for cells cultured in CM from undifferentiated preadipocytes was used for all the experiments involving melanoma cell culture.

Further, to confirm the molecular events involved in impaired response to DTIC, B16F10 and B16F1 cells were chronically grown in CM collected from undifferentiated (PA) and differentiated (ID) 3T3-L1 cells for 10 days. Using immunofluorescence staining, we observed that the level of FASN, Cav-1, and P-gp was increased in the cells cultured in CM from differentiated adipocytes in comparison to those cultured in CM from undifferentiated preadipocytes (Fig. 4b and Additional file 6: Figure S4). This suggests that altered levels of adipocyte-secreted factors are involved in modulating FASN, Cav-1, and P-gp levels which, in turn, influence the outcome of DTIC therapy in melanoma cells.

To mechanistically analyze the involvement of adipocyte-secreted factors in modulating P-gp expression and its functionality, Rh-123 efflux assay was performed. We observed that B16F10 cells grown chronically in the CM from differentiated adipocytes exhibited increased efflux of Rh-123 dye. In contrast, enhanced retention of Rh-123 was detected in B16F10 and B16F1 cells grown in CM from undifferentiated preadipocytes (Fig. 4c and Additional file 7: Figure S5). Thus, reduced uptake in B16F10 and B16F1 cells is likely a consequence of overexpression of P-gp, which is responsible for the efflux of drugs from the cells. The involvement of P-gp was further confirmed by treating B16F10 and B16F1 cells cultured in CM from differentiated adipocytes with verapamil, which increased Rh-123 retention in this setting (Fig. 4c and Additional file 7: Figure S5). These findings suggest that P-gp plays a crucial role in the impairment of chemotherapeutic response under obesity which is primarily mediated by adipocyte-secreted factors.

Differentiation of 3T3-L1 adipocytes is inhibited by orlistat, which leads to reduction in the levels of adipocyte-secreted factors [24]. We sought to determine whether the diminished level of adipocyte-secreted factors (under inhibition of adipocyte differentiation by orlistat) has any implications in the response of B16F10 cells to DTIC. We also noticed that the rate of Rh-123 retention was increased in B16F10 cells cultured in CM from orlistat-treated differentiating adipocytes (Fig. 4c and Additional file 7: Figure S5). Next, long-term colony formation assay was performed to verify whether CM obtained from either preadipocytes or differentiated adipocytes could influence the survival of melanoma cells in presence of the drug. DTIC treatment did not remarkably affect the survival of B16F10 and B16F1 cells grown in CM collected from differentiated ID adipocytes (Fig. 4d, e and Additional file 8: Figure S6). The survival of B16F10 and B16F1 cells cultured in CM from orlistat-treated differentiating adipocytes was reduced significantly to approximately 30% upon DTIC treatment (Fig. 4d, e and Additional file 8: Figure S6), suggesting that reduction in the level of secretory adipokines correlates with improved response to DTIC. Mechanistically, the sensitivity of B16F10 and B16F1 cells grown in CM collected from differentiated adipocytes to DTIC was increased upon inhibiting FASN, Cav-1, and P-gp individually (Fig. 4d, e and Additional file 8: Figure S6). Sensitivity to DTIC improved dramatically (approximately 80%) upon simultaneous treatment with all the three inhibitors (Fig. 4d, e and Additional file 8: Figure S6).

## Discussion

In the present study, we report that diet-induced obesity is associated with impaired response of melanoma to DTIC. Importantly, weight loss interventions can significantly improve the therapeutic efficacy of DTIC. As adiposity influences tumor progression, a combination treatment of obesity together with anticancer therapy is likely to improve survival [28]. Herein, we targeted adiposity and nutrient signaling together, to enhance the therapeutic efficacy of DTIC. This study provides insights on reduction in obesity by orlistat and diet restriction improves sensitivity of melanoma to DTIC, thereby prolonging survival. However, cachexia (a multifactorial wasting syndrome, characterized by the loss of adipose tissue and muscle) and severe weight loss are common in the advanced melanoma, and therefore, the adverse effects of obesity may be more important at relatively early stages of the disease. Use of orlistat as an antiobesity agent serves multiple purpose: (i) an inhibitor of lipase and hence reduction in absorption of fat by intestine [35], (ii) reported to possess anticancer property [36], and (iii) as an inhibitor of differentiation of preadipocytes to adipocytes [27].

Our study provides a preclinical evidence that the outcome of DTIC treatment is impaired in obese mice. Surprisingly, DTIC treatment instead of restricting growth actually promotes rapid tumor growth in obese mice causing decreased overall survival of tumor-bearing obese mice, as compared to untreated counterparts. This observation raises a serious question as to why DTIC treatment paradoxically promotes melanoma growth, and decreases overall survival under obese state. Earlier, it has been reported that chronic exposure of cisplatin to lung cancer cells enhances damage repair and tumor progression [37]. Moreover, Lev et al. have also shown that DTIC promotes rapid melanoma growth and metastasis by increasing the levels of IL8/VEGF [38, 39].

Our results show that levels of P-gp and other molecules involved in drug resistance such as FASN and Cav-1 are elevated in tumor excised from obese mice treated or untreated with DTIC as compared to mice kept on ND. Increase in the level of FASN and Cav-1 has been reported to be associated with development of resistance to cancer therapy [40–42]. Recently, we have shown that Akt mediates increased melanoma progression under obese state [27] and is also involved in regulating drug-resistant phenotype in several cancers [43]. Plasma membrane-associated molecule P-gp, which belongs to multidrug resistance (MDR) family of proteins, is involved in the efflux of drugs from cancer cells [44, 45]. Importantly, there is a molecular link between FASN, Cav-1, and P-gp signaling pathways which is regulated by adipokine-driven Akt activation under obese state. We have previously reported that FASN and Cav-1 interact with each other and are regulated by adipokines [23]. FASN modulates Cav-1-dependent signaling pathways and controls proliferation of cancer cells [42]. Since Cav-1 is a membrane protein and is reported to be associated with drug-resistant phenotype, it is quite likely that it might directly or indirectly influence the activity of P-gp. Owing to the enhanced activity of P-gp, tumor cells accumulate very less amount of DTIC under obese state as compared to its lean counterparts. This leads to proportional increase in the level of DTIC in plasma and accumulation in other vital organs (Additional file 9: Figure S7). Excess accumulation of DTIC in normal organs under obese state may contribute to toxicity and thus also affect overall survival.

Our in vivo and in vitro data suggest that obesity-associated factors themselves promote drug-resistant phenotype in cancer cells. Moreover, it has been reported that drug-resistant cells are highly aggressive and grow rapidly as compared to untreated counterparts [37–40]. Therefore, enhanced tumor growth owing to the DTIC treatment under obese state can be explained based on following possibilities. First, due to the accumulation of low quantity of drug in cells under obese state, cancer cells can develop drug-resistant phenotype which is typically more aggressive than untreated cells. Secondly, it has been reported that cancer cells exhibit enhanced damage repair mechanism against DNA intercalating drugs [37]. Owing to the robust DNA repair mechanism, melanoma cells can also escape from the cytotoxic effect of drugs. DTIC induces cytotoxicity in cancer cells by inducing DNA damage, which actually depends on the quantity of drug present in the nucleus. Put together, insufficient quantity of DTIC present in the cancer cells under obese condition coupled with the ability to rapidly clear DTIC-DNA adduct might promote the development of aggressive phenotype. Moreover, chromosomal abnormality due to abnormal repair mechanisms could also aggravate the melanoma growth. Yet another mechanism that could contribute to the rapid growth is enhanced secretion of IL8/VEGF by cancer cells in response to drugs [38, 39]. Furthermore, activation of Akt signaling pathway and increased level of FASN and Cav-1 contribute to drug-resistant phenotype under obese state [26, 27, 42]. Therefore, it is likely that adipokine-driven activation of signaling pathways and underexposure of drugs lead to development of highly aggressive melanoma phenotype in obese mice.

Epidemiological studies have shown that weight loss alleviates obesity-associated complications and produces disproportionate benefit to health [46]. Data from observational and calorie-restriction studies in animal models support a beneficial role for weight loss interventions in counteracting neoplasia-promoting role of obesity [22, 28]. Interestingly, by employing the strategy to control adiposity, we show that orlistat treatment or shifting from HFD to ND improves DTIC efficacy and increases overall survival of mice. And further, combining these two strategies together leads to improvement in the therapeutic outcome of DTIC and prolong survival of obese mice. Moreover, we report that when obesity was controlled by using pharmacological or dietary interventions, the levels of FASN, Cav-1, and P-gp were reduced which correlates with improvement in the effectiveness of DTIC on ectopic tumors in mice. Interestingly, weight control intervention profoundly increases the accumulation of DTIC in tumor and subsequently reduces its level in plasma and other tissues. These findings suggest that controlling adiposity/body weight is beneficial towards the outcome of chemotherapy and may contribute towards reduction in generalized toxicity to organs. Additionally, this may also be helpful in preventing the development of drug-resistant phenotype in cancers.

Studies from in vitro and in vivo results conclude that stored fat, and adipokines, the adipocyte-derived factors or hormones, aid in the progression of melanoma by up-regulating and driving multiple etiological pathways and regulating signaling cascades [47–49]. Adipokines can specifically trigger several distinct transcriptional programs, those that promote tumorigenesis, cell survival and proliferation, and angiogenesis and invasiveness [50–52]. Altered secretion profile of leptin, and adiponectin, and other inflammatory adipokines such as IL-6 and TNF-α, by interacting with signaling network, accelerate neoplasia [13]. These factors by enhancing P-gp may promote drug efflux, causing accumulation of drug in plasma and induce toxicity to vital organs. In addition, the nutrients and net available energy are also crucial factors that decide the fate of both normal and malignant cells. However, assessing net energy intake and expenditure is difficult to evaluate in humans. Nutrient signaling is an integral part of metabolic regulatory network and links nutrient availability with cell growth and proliferation. Therefore, it is rational to target adipose tissue with antiobesity drug and energy restriction simultaneously.

Using unique strategy by growing melanoma cells in either serum collected from obese mice treated or untreated with DTIC or orlistat, as well as in conditioned medium collected from adipocytes, we show that adipocyte secretory factors actually modulate the therapeutic response of melanoma cells to DTIC. Adipocytes are also known to protect leukemia and breast cancer cells from chemotherapy and radiotherapy, respectively [53, 54]. On the similar line, our study demonstrates that melanoma cells grown in the presence of serum collected from obese mice as well as in the CM collected from differentiated adipocytes cause an increase in IC_50_ value of DTIC, suggesting that adipocyte-secreted factors adversely affect the outcome of chemotherapy. Melanoma cells grown under these conditions develop chemo-resistant phenotype, characterized by increased expression of FASN, Cav-1, and P-gp. Inhibition of these molecules reverses the chemo-resistant phenotype induced by adipocyte secretory factors. These data imply that adipocyte-secreted factors are crucial in modulating important tumor-promoting molecules which, in turn, affect the outcome of cancer therapy. Collectively, these results suggest that, under obese state, melanoma cells in addition to acquiring a drug-resistant phenotype also develop aggressive proliferative phenotype, as compared to untreated counterparts.

## Conclusions

**Fig. 5:**
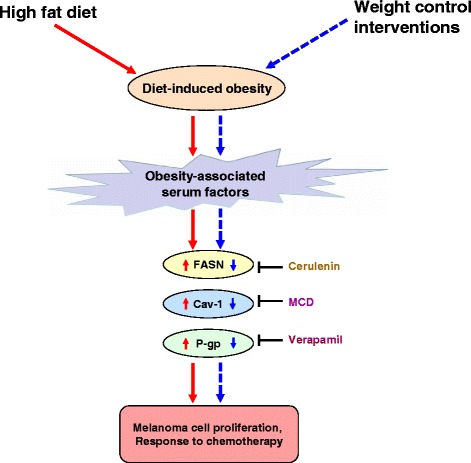
Proposed model of impact of obesity and weight control interventions on the outcome of dacarbazine therapy in melanoma. Obesity-associated factors impair the response of melanoma to DTIC by enhancing the levels of tumor-promoting molecules (*solid red arrow marks*). On the other hand, controlling obesity through weight interventions causes normalization in obesity-associated factors. This in turn improves the efficacy of DTIC through reducing in the levels of melanoma cell growth promoting molecules (*dotted blue arrow marks*)

This study provides a mechanistic link between obesity and the outcome of chemotherapy in melanoma through involvement of obesity-associated factors, which alter the status of key signaling molecules involved in cell survival/proliferation, drug efflux, and drug resistance. Interventions leading to weight loss or prevention of weight gain restrict melanoma growth (see the schematic overview in Fig. 5). We investigated the impact of obesity in the outcome of chemotherapy only in a mouse melanoma tumor isograft model, so our findings justify further clinical and experimental research.

## Additional files

Additional file 1: Table S1. Composition of diets used in the study. Normal diet (ND) was procured from Amrut Laboratory Animal Feed, Pune, India, and high fat diet (HFD) was purchased from Provimi Animal Nutrition Pvt. Ltd., Bangalore, India. *, HFD was also supplemented with 400 g groundnut and 200 g dried coconut per kg body weight of mice. (PDF 169 kb)
Additional file 2: Table S2. Obesity-associated parameters in the experimental ND mice recorded at the end of the experiment. ND male C57BL/6J mice were divided into two major groups. One group was orally treated with orlistat every alternate day before inoculating with B16F10 cells. The second group was orally given vehicle control on every alternate day for 8 weeks. Mice from both the groups (*N* = 11 per each group) were injected (s.c.) with B16F10 cells (2 × 10^5^ cells/mouse in 100 μl PBS). After tumor formation, vehicle or DTIC treatment was given as per the experimental layout shown in Figure 1. Their body weight was monitored weekly throughout the study, and serum was collected at the end of the experiment. Blood glucose, serum TG, serum cholesterol, serum-free fatty acids, and serum LDLc were measured. Serum factors including leptin, adiponectin, insulin, resistin, IL-6, and TNF-α were estimated by ELISA. The results are given as means ± standard deviation. (PDF 113 kb)
Additional file 3: Figure S1. Effect of obesity-associated serum factors on the protein level of P-gp, Cav-1, and FASN in B16F1 cells. B16F1 cells were chronically grown in medium containing 5% serum collected from ND or HFD C57BL/6J mice for 15 days. Thereafter, these cells were subjected to immunofluorescence confocal staining of the indicated molecules. The data were recorded using Zeiss LSM510 META Confocal Microscope. (Scale bar = 20 μm). (PDF 189 kb)
Additional file 4: Figure S2. Effect of obesity-associated serum factors on rhodamine-123 (Rh-123) efflux in B16F1 cells. B16F1 cells were chronically grown in medium containing 5% serum collected from ND or HFD C57BL/6J mice for 15 days. Thereafter, these cells were subjected to Rh-123 efflux assay. Data were acquired on FACS Calibur and analyzed using BD CellQuest Pro software. The data are representative of experiments performed three times. (PDF 120 kb)
Additional file 5: Figure S3. Effect of inhibition of FASN, Cav-1, and P-gp on response of B16F1 cells to DTIC. B16F1 cells were chronically grown in medium containing 5% serum collected from experimental ND or HFD C57BL/6J mice for 15 days. Thereafter, these cells were subjected to long-term survival assay. First, cells were treated with respective inhibitors followed by treatment of DTIC for 48 h. Then, the medium was changed and fresh medium was added. The medium was changed every 2–3 days. After 10 days, the cells were stained with 0.05% crystal violet and images were taken using Olympus digital camera. Data were quantitated using ImageJ software. The data are representative of experiments performed three times; Ceru or C = cerulenin; MCD or M = methyl β-cyclodextrin; Vera or V = verapamil. The results are given as means ± standard deviation; *, *p* < 0.05. (PDF 666 kb)
Additional file 6: Figure S4. Effect of adipocyte-secreted factors on the protein level of P-gp, Cav-1, and FASN in B16F1 cells. 3T3-L1 cells were induced to differentiate with 500 μM 3-isobutyl-1-methylxanthine (IBMX) and 250 μM dexamethasone (DEX). The medium was changed every alternate day. After 10 days, cells were washed twice with DMEM and fresh DMEM without serum was added to the cells. After 18 h, conditioned medium (CM) was collected from undifferentiated or differentiated 3T3-L1 cells. Thereafter, B16F1 cells were cultured in these CM for 48 h, and these cells were subjected to immunofluorescence confocal staining for the indicated molecules. The data were recorded using Zeiss LSM510 META Confocal Microscope (Scale bar = 20 μm); PA = preadipocytes; ID = differentiated 3T3-L1 cells induced to differentiate by IBMX and DEX. (PDF 215 kb)
Additional file 7: Figure S5. Effect of adipocyte-secreted factors on Rh-123 efflux in B16F1 cells. 3T3-L1 cells were induced to differentiate with 500 μM 3-isobutyl-1-methylxanthine (IBMX) and 250 μM dexamethasone (DEX). The medium was changed every alternate day. After 10 days, cells were washed twice with DMEM and fresh DMEM without serum was added to the cells. After 18 h, conditioned medium (CM) was collected from undifferentiated or differentiated 3T3-L1 cells. Thereafter, B16F10 or B16F1 cells were cultured in these CM for 48 h. Further, these cells were subjected to Rh-123 efflux assay. Data were acquired on FACS Calibur and analyzed using BD CellQuest Pro software. The data are representative of experiments performed three times; PA = preadipocytes; ID = differentiated 3T3-L1 cells induced by IBMX and DEX; Vera = verapamil. (PDF 150 kb)
Additional file 8: Figure S6. Effect of inhibiting FASN, Cav-1, and P-gp on response of B16F1 cells to DTIC upon culture in CM collected from 3T3-L1 cells. 3T3-L1 cells were induced to differentiate with 500 μM 3-isobutyl-1-methylxanthine (IBMX) and 250 μM dexamethasone (DEX). The medium was changed every alternate day. After 10 days, cells were washed twice with DMEM and fresh DMEM without serum was added to the cells. After 18 h, conditioned medium (CM) was collected from undifferentiated or differentiated 3T3-L1 cells. Thereafter, B16F10 or B16F1 cells were cultured in these CM for 48 h. First, cells were treated with respective inhibitors followed by treatment of DTIC for 48 h. Then, the medium was changed and fresh medium was added. The medium was changed every 2–3 days. After 10 days, the cells were stained with 0.05% crystal violet and images were taken using Olympus digital camera. Data were quantitated using ImageJ software. The data are representative of experiments performed three times; PA = preadipocytes; ID = differentiated 3T3-L1 cells induced by IBMX and DEX; Ceru or C = cerulenin; MCD or M = methyl β-cyclodextrin; Vera or V = verapamil. The results are given as means ± standard deviation; *, *p* < 0.05. (PDF 538 kb)
Additional file 9: Figure S7. MS analysis on the distribution of DTIC in tumors and organ samples collected from experimental ND or HFD mice. Lysates of tumors and organ samples from the experimental ND or HFD mice were prepared and subjected to liquid chromatography-electrospray ionization tandem mass spectrometry (LC-ESI MS/MS). The spectrum shows protonated mass of DTIC at 183.0984, m/z and the product ions at m/z, 166.0733, 138.0396, and 123.0427 (similar fragmentation patterns were observed in tumors and tissue samples of mice treated with DTIC). The inset contains chemical structure of DTIC with the fragmentation patterns marked. All the MS data were collected in the presence of internal standards, where mass tolerance of DTIC was maintained well within 3 ppm. (PDF 122 kb)

## Abbreviations

Cav-1: Caveolin-1
DTIC: Dacarbazine
FASN: Fatty acid synthase
HFD: High fat diet
ND: Normal diet
P-gp: P-glycoprotein
