# Supplementary material for: Weight control interventions improve therapeutic efficacy of dacarbazine in melanoma by reversing obesity-induced drug resistance
Source: Cancer Metab. 2016 Dec 7;4:21. doi: 10.1186/s40170-016-0162-8 (PMC5142287; doi:10.1186/s40170-016-0162-8)
Supplement: Additional file 1: Table S1. — Composition of diets used in the study. Normal diet (ND) was procured from Amrut Laboratory Animal Feed, Pune, India, and high fat diet (HFD) was purchased from Provimi Animal Nutrition Pvt. Ltd., Bangalore, India. *, HFD was also supplemented with 400 g groundnut and 200 g dried coconut per kg body weight of mice. (PDF 169 kb) [file 40170_2016_162_MOESM1_ESM.pdf]

Additional File 1: Table S1.

Ingredients	Normal diet (ND)	High fat diet (HFD)*
Crude protein	19.26%	22.55%
Crude fat	4.20%	24.18%
Crude carbohydrate	56.00%	35.00%
Crude fiber	3.00%	2.98%
Calcium	2.15%	1.89%
Phosphorus	1.10%	0.88%
Moisture	8.50%	3.44%
Total ash	7.00%	8.66%

Diet	ND	HFD	Groundnuts	Dry-coconuts
**Energy Kcal/g**	3.55 Kcal/g	4.49 Kcal/g	5.67 Kcal/g	1.87 Kcal/g

Table S1 Malvi et al. 2016

Table S1. Composition of diets used in the study. Normal diet (ND) was procured form Amrut Laboratory Animal Feed, Pune, India and high fat diet (HFD) was purchased from Provimi Animal Nutrition Pvt. Ltd., Bangalore, India. *, HFD was also supplemented with 400 g groundnut and 200 g dried coconut per kg body weight of mice.
