# Supplementary material for: Weight control interventions improve therapeutic efficacy of dacarbazine in melanoma by reversing obesity-induced drug resistance
Source: Cancer Metab. 2016 Dec 7;4:21. doi: 10.1186/s40170-016-0162-8 (PMC5142287; doi:10.1186/s40170-016-0162-8)
Supplement: Additional file 2: Table S2. — Obesity-associated parameters in the experimental ND mice recorded at the end of the experiment. ND male C57BL/6J mice were divided into two major groups. One group was orally treated with orlistat every alternate day before inoculating with B16F10 cells. The second group was orally given vehicle control on every alternate day for 8 weeks. Mice from both the groups (N = 11 per each group) were injected (s.c.) with B16F10 cells (2 × 10^5^ cells/mouse in 100 μl PBS). After tumor formation, vehicle or DTIC treatment was given as per the experimental layout shown in Figure 1. Their body weight was monitored weekly throughout the study, and serum was collected at the end of the experiment. Blood glucose, serum TG, serum cholesterol, serum-free fatty acids, and serum LDLc were measured. Serum factors including leptin, adiponectin, insulin, resistin, IL-6, and TNF-α were estimated by ELISA. The results are given as means ± standard deviation. (PDF 113 kb) [file 40170_2016_162_MOESM2_ESM.pdf]

## Additional File2: Table S2:

## Table: Evaluation of obesity-associated factors in ND C57BL/6J mice

| Parameters | C57BL/6J ND | | | |
|---|---|---|---|---|
| | ND-Ctrl | ND-DTIC | ND-Orli | ND-Orli DTIC |
| Body weight (g) | 21.34 ± 1.88 | 21. 15 ± 1.81 | 21.50 ± 1.66 | 21.71 ± 1.85 |
| Blood glucose (mg/dl) | 164.23 ± 7.23 | 161.17 ± 5.81 | 161.83 ± 5.49 | 162.00 ± 6.63 |
| Serum TG (mg/dl) | 54.94 ± 3.39 | 55.40 ± 3.54 | 54.93 ± 3.64 | 55.00 ± 3.44 |
| Serum cholesterol (mg/dl) | 81.64 ± 6.64 | 82.82 ± 7.04 | 81.36 ± 6.86 | 80.81 ± 6.19 |
| Serum LDLc (mg/dl) | 52.11 ± 3.82 | 53.12 ± 4.11 | 51.26 ± 4.31 | 50.80 ± 5.05 |
| Serum free fatty acids (mM/L) | 0.89 ± 0.05 | 0.90 ± 0.05 | 0.89 ± 0.04 | 0.89 ± 0.04 |
| Serum leptin (ng/ml) | 15. 73 ± 2.41 | 15.74 ± 2.72 | 15.43 ± 3.06 | 15.53 ± 2.53 |
| Serum adiponectin (ng/ml) | 14420 ± 667 | 13873 ± 836 | 13803 ± 561 | 14340 ± 739 |
| Serum insulin (μg/L) | 0.1210 ± 0.011 | 0.1143 ± 0.011 | 0.1156 ± 0.006 | 0.1208 ± 0.005 |
| Serum resistin (ng/ml) | 0.99 ± 0.04 | 0.99 ± 0.06 | 0.98 ± 0.06 | 0.99 ± 0.05 |
| Serum IL-6 (pg/ml) | 20.20 ± 1.02 | 20.19 ± 2.13 | 19.99 ± 0.67 | 19.82 ± 1.31 |
| Serum TNF-α (pg/ml) | 11.96 ± 1.46 | 12.35 ± 1.24 | 11.78 ± 0.95 | 12.02 ± 1.22 |

***Table S2 Malvi et al. 2016***

**Table S2.** Obesity-associated parameters in the experimental ND mice recorded at the end of the experiment. ND male C57BL/6J mice were divided into two major groups. One group was orally treated with orlistat every alternate day before inoculating with B16F10 cells. The second group was orally given vehicle control on every alternate day for 8 weeks. All the

groups of mice (N = 11 per each group) were injected (s.c.) with B16F10 cells ($2 \times 10^5$ cells/mouse in 100 µl PBS). After tumor formation, vehicle or DTIC treatment was given as per the experimental layout shown in Figure 1. Their body weight was monitored weekly throughout the study and serum was collected at the end of the experiment. Blood glucose, serum TG, serum cholesterol, serum free fatty acids and serum LDLc were measured. Serum factors including leptin, adiponectin, insulin, resistin, IL-6 and TNF-α were estimated by ELISA. The results are given as means ± standard deviation.
