# Supplementary material for: Weight control interventions improve therapeutic efficacy of dacarbazine in melanoma by reversing obesity-induced drug resistance
Source: Cancer Metab. 2016 Dec 7;4:21. doi: 10.1186/s40170-016-0162-8 (PMC5142287; doi:10.1186/s40170-016-0162-8)
Supplement: Additional file 3: Figure S1. — Effect of obesity-associated serum factors on the protein level of P-gp, Cav-1, and FASN in B16F1 cells. B16F1 cells were chronically grown in medium containing 5% serum collected from ND or HFD C57BL/6J mice for 15 days. Thereafter, these cells were subjected to immunofluorescence confocal staining of the indicated molecules. The data were recorded using Zeiss LSM510 META Confocal Microscope. (Scale bar = 20 μm). (PDF 189 kb) [file 40170_2016_162_MOESM3_ESM.pdf]

Additional File 3: Figure S1:

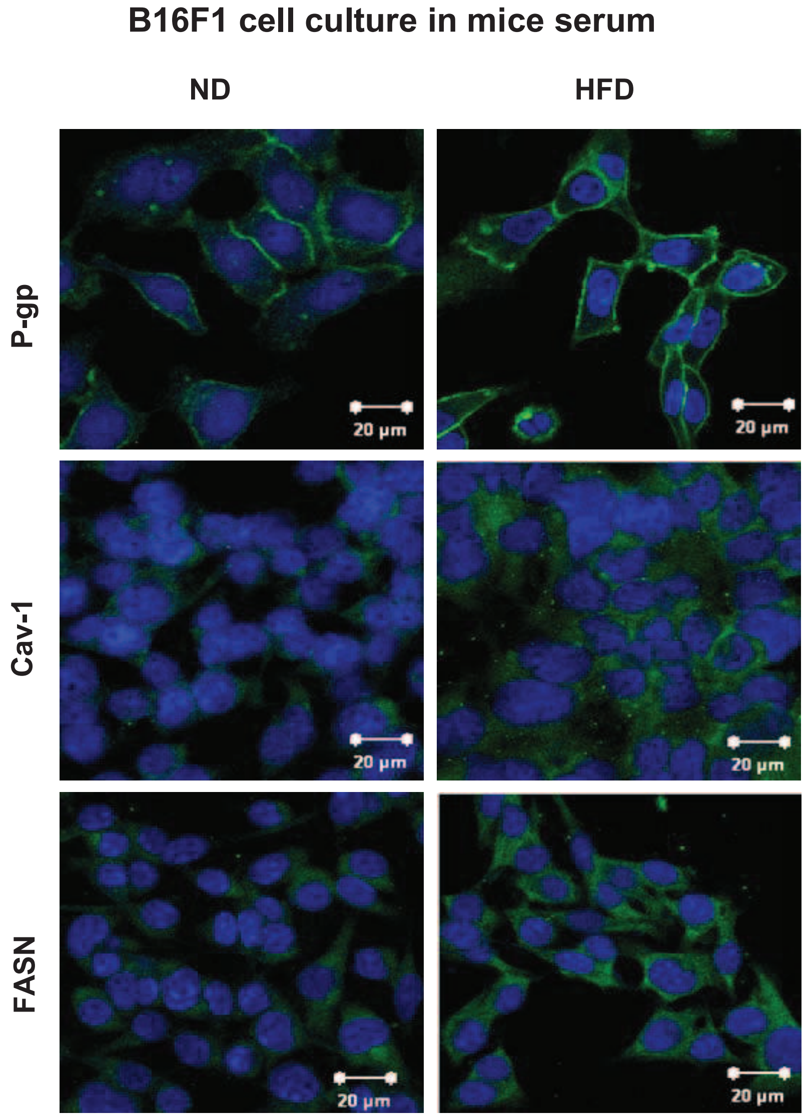

Figure S1 Malvi et al. 2016

Figure S1. Effect of obesity-associated serum factors on the protein level of P-gp, Cav-1 and FASN in B16F1 cells. B16F1 cells were chronically grown in medium containing 5% serum collected from ND or HFD C57BL/6J mice for 15 days. Thereafter, these cells were subjected to immunofluorescence confocal staining of the indicated molecules. The data were recorded using Zeiss LSM510 META Confocal Microscope. (Scale bar = 20 µm).
