# Supplementary material for: Weight control interventions improve therapeutic efficacy of dacarbazine in melanoma by reversing obesity-induced drug resistance
Source: Cancer Metab. 2016 Dec 7;4:21. doi: 10.1186/s40170-016-0162-8 (PMC5142287; doi:10.1186/s40170-016-0162-8)
Supplement: Additional file 4: Figure S2. — Effect of obesity-associated serum factors on rhodamine-123 (Rh-123) efflux in B16F1 cells. B16F1 cells were chronically grown in medium containing 5% serum collected from ND or HFD C57BL/6J mice for 15 days. Thereafter, these cells were subjected to Rh-123 efflux assay. Data were acquired on FACS Calibur and analyzed using BD CellQuest Pro software. The data are representative of experiments performed three times. (PDF 120 kb) [file 40170_2016_162_MOESM4_ESM.pdf]

Additional File 4: Figure S2:

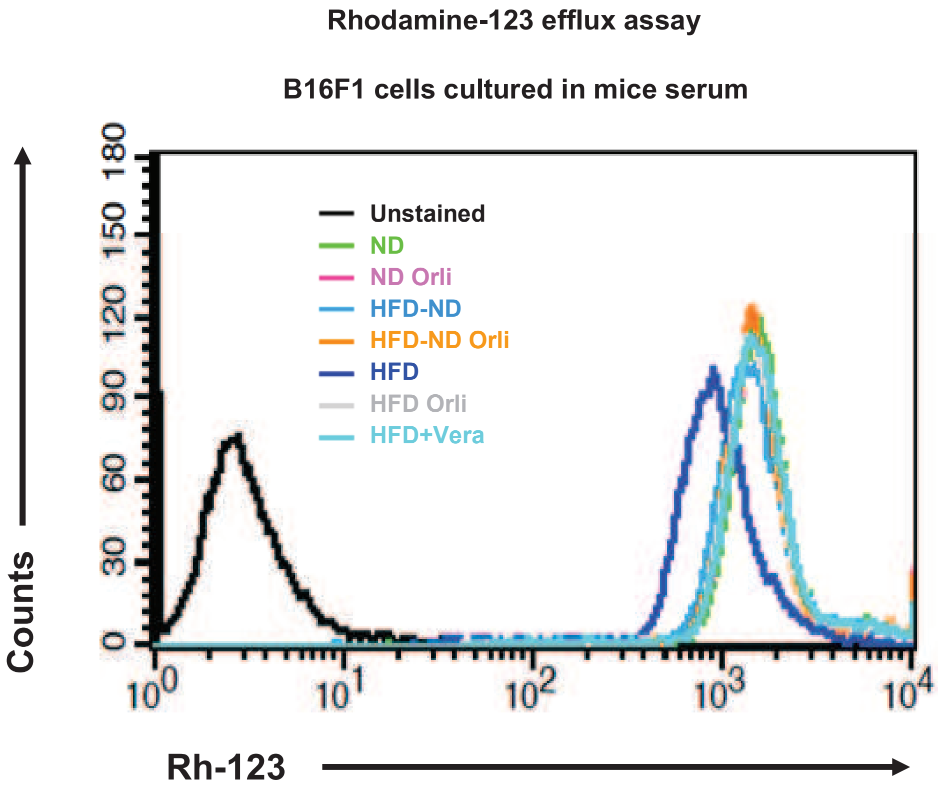

Figure S2 Malvi et al. 2016

Figure S2. Effect of obesity-associated serum factors on rhodamine-123 (Rh-123) efflux in B16F1 cells. B16F1 cells were chronically grown in medium containing 5% serum collected from ND or HFD C57BL/6J mice for 15 days. Thereafter, these cells were subjected to Rh-123 efflux assay. Data were acquired on FACS Calibur and analyzed using BD CellQuest Pro software. The data are representative of experiments performed three times.
