# Supplementary material for: Weight control interventions improve therapeutic efficacy of dacarbazine in melanoma by reversing obesity-induced drug resistance
Source: Cancer Metab. 2016 Dec 7;4:21. doi: 10.1186/s40170-016-0162-8 (PMC5142287; doi:10.1186/s40170-016-0162-8)
Supplement: Additional file 5: Figure S3. — Effect of inhibition of FASN, Cav-1, and P-gp on response of B16F1 cells to DTIC. B16F1 cells were chronically grown in medium containing 5% serum collected from experimental ND or HFD C57BL/6J mice for 15 days. Thereafter, these cells were subjected to long-term survival assay. First, cells were treated with respective inhibitors followed by treatment of DTIC for 48 h. Then, the medium was changed and fresh medium was added. The medium was changed every 2–3 days. After 10 days, the cells were stained with 0.05% crystal violet and images were taken using Olympus digital camera. Data were quantitated using ImageJ software. The data are representative of experiments performed three times; Ceru or C = cerulenin; MCD or M = methyl β-cyclodextrin; Vera or V = verapamil. The results are given as means ± standard deviation; *, p < 0.05. (PDF 666 kb) [file 40170_2016_162_MOESM5_ESM.pdf]

Additional File 5: Figure S3:

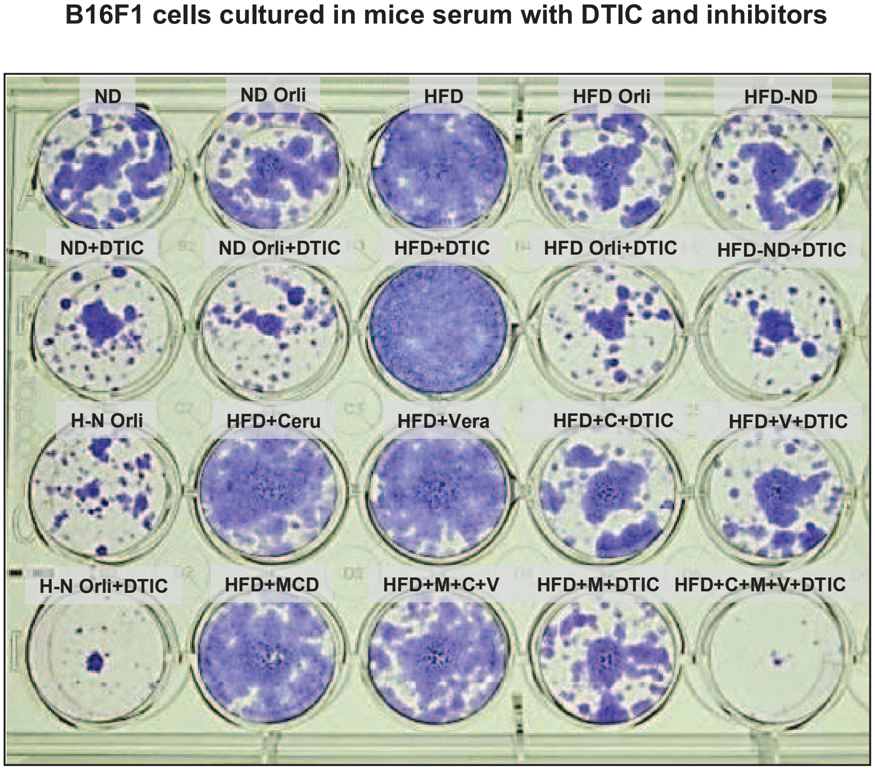

Figure S3 Malvi et al. 2016

Figure S3. Effect of inhibition of FASN, Cav-1 and P-gp on response of B16F1 cells to DTIC. B16F1 cells were chronically grown in medium containing 5% serum collected from experimental ND or HFD C57BL/6J mice for 15 days. Thereafter, these cells were subjected to long term survival assay. First, cells were treated with respective inhibitors followed by treatment of DTIC for 48 h. Then, the medium was changed and fresh medium was added. The medium was changed every 2-3 days. After 10 days, the cells were stained with 0.05% crystal violet and images were taken using Olympus digital camera. Data were quantitated using Image J software. The data are representative of experiments performed three times; Ceru or C = cerulenin; MCD or M = methyl β-cyclodextrin; Vera or V = verapamil. The results are given as means ± standard deviation; *, $p < 0.05$.
