# Supplementary material for: Weight control interventions improve therapeutic efficacy of dacarbazine in melanoma by reversing obesity-induced drug resistance
Source: Cancer Metab. 2016 Dec 7;4:21. doi: 10.1186/s40170-016-0162-8 (PMC5142287; doi:10.1186/s40170-016-0162-8)
Supplement: Additional file 6: Figure S4. — Effect of adipocyte-secreted factors on the protein level of P-gp, Cav-1, and FASN in B16F1 cells. 3T3-L1 cells were induced to differentiate with 500 μM 3-isobutyl-1-methylxanthine (IBMX) and 250 μM dexamethasone (DEX). The medium was changed every alternate day. After 10 days, cells were washed twice with DMEM and fresh DMEM without serum was added to the cells. After 18 h, conditioned medium (CM) was collected from undifferentiated or differentiated 3T3-L1 cells. Thereafter, B16F1 cells were cultured in these CM for 48 h, and these cells were subjected to immunofluorescence confocal staining for the indicated molecules. The data were recorded using Zeiss LSM510 META Confocal Microscope (Scale bar = 20 μm); PA = preadipocytes; ID = differentiated 3T3-L1 cells induced to differentiate by IBMX and DEX. (PDF 215 kb) [file 40170_2016_162_MOESM6_ESM.pdf]

Additional File 6: Figure S4:

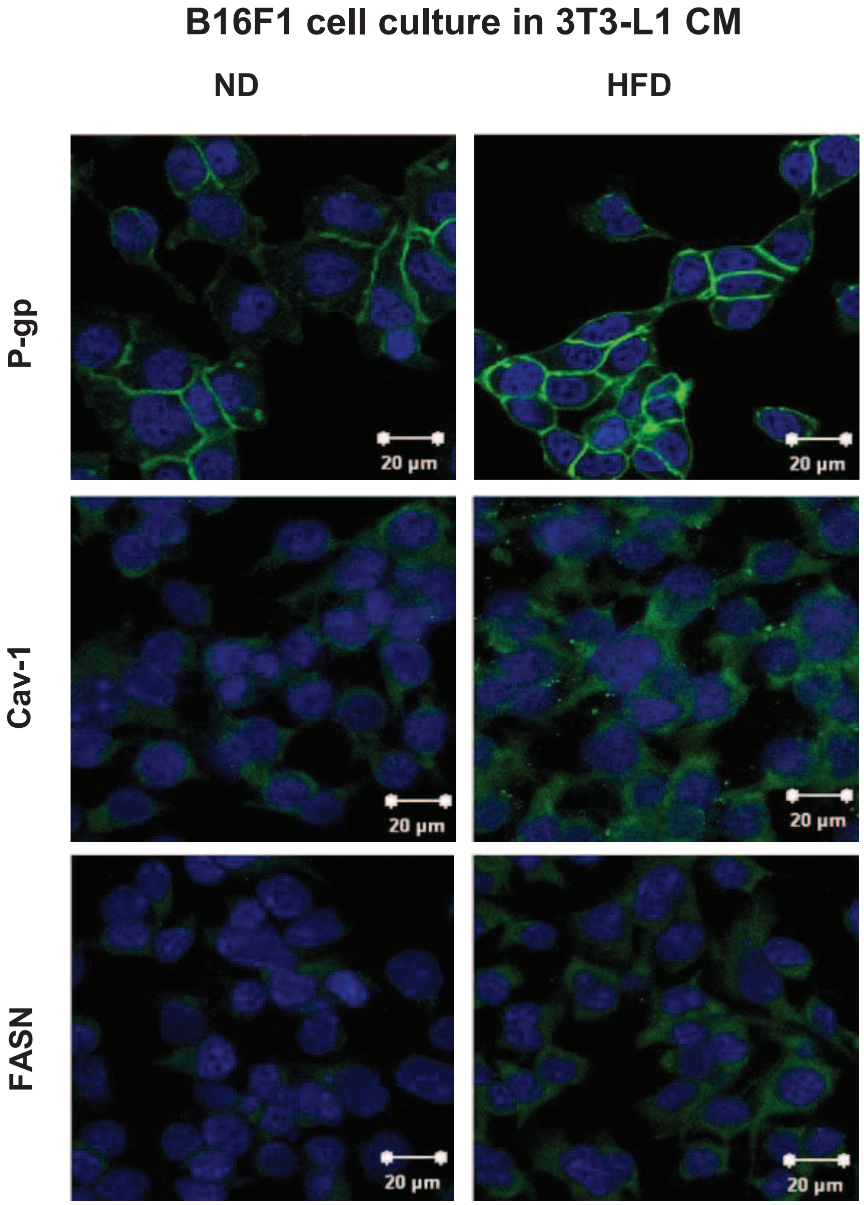

Figure S4 Malvi et al. 2016

Figure S4. Effect of adipocyte-secreted factors on the protein level of P-gp, Cav-1 and FASN in B16F1 cells. 3T3-L1 cells were induced to differentiate with 500 μM 3-isobutyl-1-methylxanthine (IBMX) and 250 μM dexamethasone (DEX). The medium was changed

every alternate day. After 10 days, cells were washed twice with DMEM and fresh DMEM without serum was added to the cells. After 18 h, conditioned medium (CM) was collected from undifferentiated or differentiated 3T3-L1 cells. Thereafter, B16F1 cells were cultured in these CM for 48 h, and these cells were subjected to immunofluorescence confocal staining for the indicated molecules. The data were recorded using Zeiss LSM510 META Confocal Microscope (Scale bar = 20 μm); PA = preadipocytes; ID = differentiated 3T3-L1 cells induced to differentiate by IBMX and DEX.
