# Supplementary material for: Weight control interventions improve therapeutic efficacy of dacarbazine in melanoma by reversing obesity-induced drug resistance
Source: Cancer Metab. 2016 Dec 7;4:21. doi: 10.1186/s40170-016-0162-8 (PMC5142287; doi:10.1186/s40170-016-0162-8)
Supplement: Additional file 7: Figure S5. — Effect of adipocyte-secreted factors on Rh-123 efflux in B16F1 cells. 3T3-L1 cells were induced to differentiate with 500 μM 3-isobutyl-1-methylxanthine (IBMX) and 250 μM dexamethasone (DEX). The medium was changed every alternate day. After 10 days, cells were washed twice with DMEM and fresh DMEM without serum was added to the cells. After 18 h, conditioned medium (CM) was collected from undifferentiated or differentiated 3T3-L1 cells. Thereafter, B16F10 or B16F1 cells were cultured in these CM for 48 h. Further, these cells were subjected to Rh-123 efflux assay. Data were acquired on FACS Calibur and analyzed using BD CellQuest Pro software. The data are representative of experiments performed three times; PA = preadipocytes; ID = differentiated 3T3-L1 cells induced by IBMX and DEX; Vera = verapamil. (PDF 150 kb) [file 40170_2016_162_MOESM7_ESM.pdf]

Additional File 7: Figure S5:

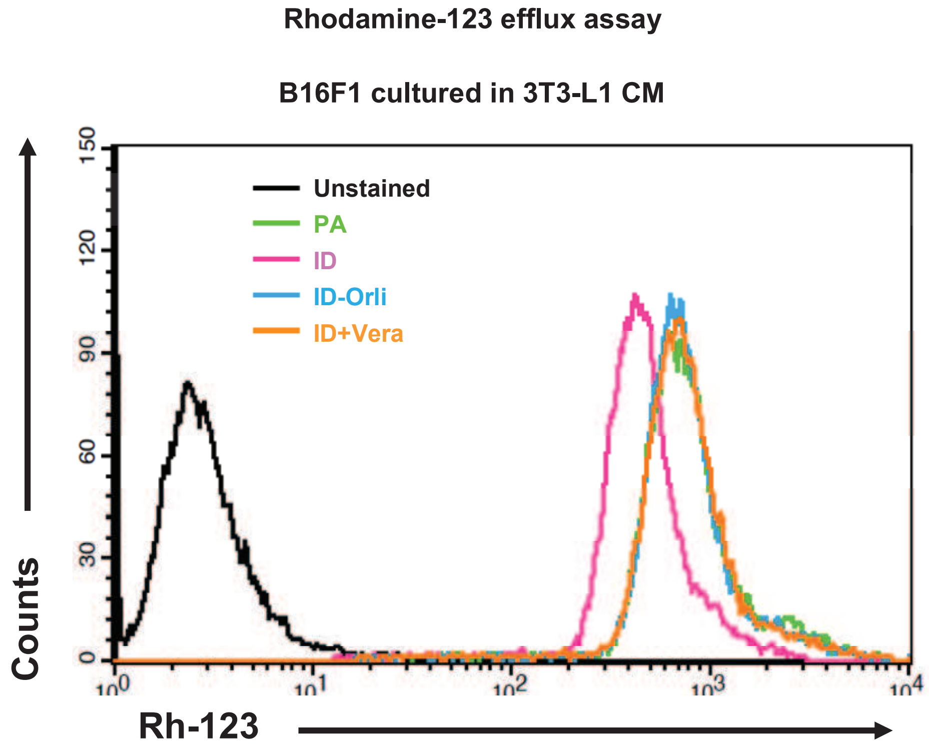

Figure S5 Malvi et al. 2016

Figure S5. Effect of adipocyte-secreted factors on Rh-123 efflux in B16F1 cells. 3T3-L1 cells were induced to differentiate with 500 µM 3-isobutyl-1-methylxanthine (IBMX) and 250 µM dexamethasone (DEX). The medium was changed every alternate day. After 10 days, cells were washed twice with DMEM and fresh DMEM without serum was added to the cells. After 18 h, conditioned medium (CM) was collected from undifferentiated or differentiated 3T3-L1 cells. Thereafter, B16F10 or B16F1 cells were cultured in these CM for 48 h. Further, these cells were subjected to Rh-123 efflux assay. Data were acquired on FACS Calibur and analyzed using BD CellQuest Pro software. The data are representative of experiments performed three times; PA = preadipocytes; ID = differentiated 3T3-L1 cells induced by IBMX and DEX; Vera = verapamil.
