# Supplementary material for: Weight control interventions improve therapeutic efficacy of dacarbazine in melanoma by reversing obesity-induced drug resistance
Source: Cancer Metab. 2016 Dec 7;4:21. doi: 10.1186/s40170-016-0162-8 (PMC5142287; doi:10.1186/s40170-016-0162-8)
Supplement: Additional file 8: Figure S6. — Effect of inhibiting FASN, Cav-1, and P-gp on response of B16F1 cells to DTIC upon culture in CM collected from 3T3-L1 cells. 3T3-L1 cells were induced to differentiate with 500 μM 3-isobutyl-1-methylxanthine (IBMX) and 250 μM dexamethasone (DEX). The medium was changed every alternate day. After 10 days, cells were washed twice with DMEM and fresh DMEM without serum was added to the cells. After 18 h, conditioned medium (CM) was collected from undifferentiated or differentiated 3T3-L1 cells. Thereafter, B16F10 or B16F1 cells were cultured in these CM for 48 h. First, cells were treated with respective inhibitors followed by treatment of DTIC for 48 h. Then, the medium was changed and fresh medium was added. The medium was changed every 2–3 days. After 10 days, the cells were stained with 0.05% crystal violet and images were taken using Olympus digital camera. Data were quantitated using ImageJ software. The data are representative of experiments performed three times; PA = preadipocytes; ID = differentiated 3T3-L1 cells induced by IBMX and DEX; Ceru or C = cerulenin; MCD or M = methyl β-cyclodextrin; Vera or V = verapamil. The results are given as means ± standard deviation; *, p < 0.05. (PDF 538 kb) [file 40170_2016_162_MOESM8_ESM.pdf]

Additional File 8: Figure S6:

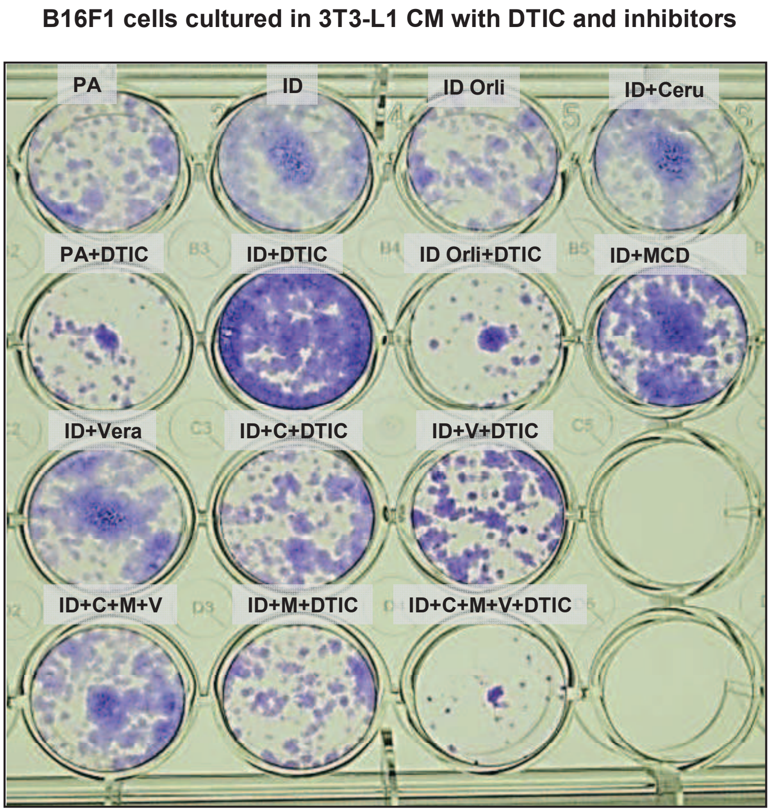

Figure S6 Malvi et al. 2016

Figure S6. Effect of inhibiting FASN, Cav-1 and P-gp on response of B16F1 cells to DTIC upon culture in CM collected from 3T3-L1 cells. 3T3-L1 cells were induced to differentiate with 500 μM 3-isobutyl-1-methylxanthine (IBMX) and 250 μM dexamethasone (DEX). The medium was changed every alternate day. After 10 days, cells were washed twice with DMEM and fresh DMEM without serum was added to the cells. After 18 h, conditioned medium (CM) was collected from undifferentiated or differentiated 3T3-L1 cells. Thereafter, B16F10 or B16F1 cells were cultured in these CM for 48 h. First, cells were treated with respective inhibitors followed by treatment of DTIC for 48 h. Then, the medium was changed

and fresh medium was added. The medium was changed every 2-3 days. After 10 days, the cells were stained with 0.05% crystal violet and images were taken using Olympus digital camera. Data were quantitated using Image J software. The data are representative of experiments performed three times; PA = preadipocytes; ID = differentiated 3T3-L1 cells induced by IBMX and DEX; Ceru or C = cerulenin; MCD or M = methyl β-cyclodextrin; Vera or V = verapamil. The results are given as means ± standard deviation; *, $p < 0.05$.
