# Supplementary material for: Weight control interventions improve therapeutic efficacy of dacarbazine in melanoma by reversing obesity-induced drug resistance
Source: Cancer Metab. 2016 Dec 7;4:21. doi: 10.1186/s40170-016-0162-8 (PMC5142287; doi:10.1186/s40170-016-0162-8)
Supplement: Additional file 9: Figure S7. — MS analysis on the distribution of DTIC in tumors and organ samples collected from experimental ND or HFD mice. Lysates of tumors and organ samples from the experimental ND or HFD mice were prepared and subjected to liquid chromatography-electrospray ionization tandem mass spectrometry (LC-ESI MS/MS). The spectrum shows protonated mass of DTIC at 183.0984, m/z and the product ions at m/z, 166.0733, 138.0396, and 123.0427 (similar fragmentation patterns were observed in tumors and tissue samples of mice treated with DTIC). The inset contains chemical structure of DTIC with the fragmentation patterns marked. All the MS data were collected in the presence of internal standards, where mass tolerance of DTIC was maintained well within 3 ppm. (PDF 122 kb) [file 40170_2016_162_MOESM9_ESM.pdf]

Additional File 9: Figure S7:

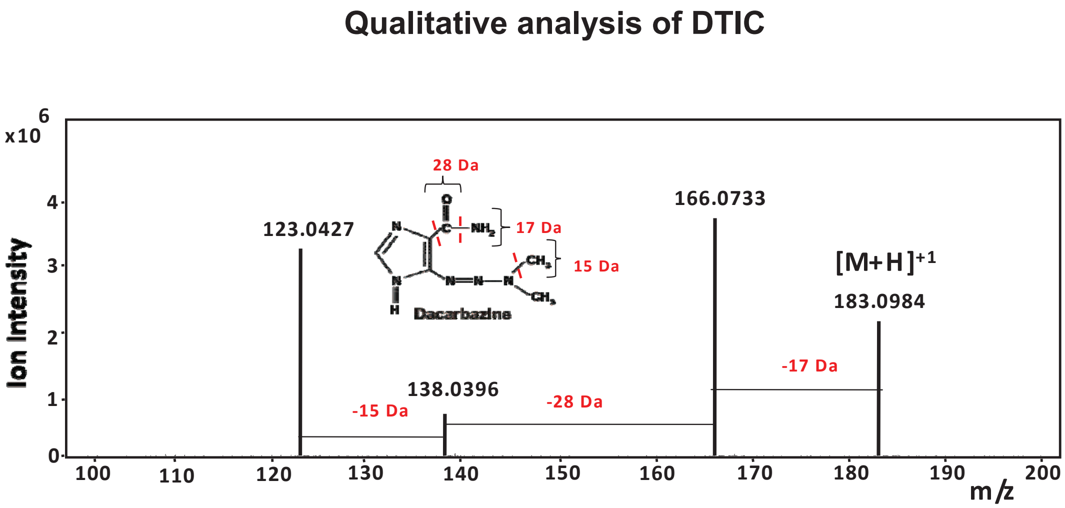

Figure S7Malvi et al. 2016

Figure S7. MS analysis on the distribution of DTIC in tumors and organ samples collected from experimental ND or HFD mice. Lysates of tumors and organ samples from the experimental ND or HFD mice were prepared and subjected to liquid chromatography-electrospray ionization tandem mass spectrometry (LC- ESI MS/MS). The spectrum shows protonated mass of DTIC at 183.0984, m/z and the product ions at m/z, 166.0733, 138.0396 and 123.0427 (similar fragmentation patterns were observed in tumors and tissue samples of mice treated with DTIC). The inset contains chemical structure of DTIC with the fragmentation patterns marked. All the MS data were collected in the presence of internal standards, where mass tolerance of DTIC was maintained well within 3 ppm.
